# Supplementary material for: Quantum-limited stochastic optical neural networks operating at a few quanta per activation
Source: Nat Commun. 2025 Jan 3;16:359. doi: 10.1038/s41467-024-55220-y (PMC11698857; doi:10.1038/s41467-024-55220-y)
Supplement: Supplementary file 1 — Supplementary Information [file 41467_2024_55220_MOESM1_ESM.pdf]

Supplementary Information for

Quantum-limited stochastic optical neural networks operating at a few quanta per activation

Shi-Yuan Ma,[1,*] Tianyu Wang,[1] Jérémie Laydevant,[1,2] Logan G. Wright,[1,3,†] and Peter L. McMahon[1,4,‡]

[1]*School of Applied and Engineering Physics, Cornell University, Ithaca, NY 14853, USA*

[2]*USRA Research Institute for Advanced Computer Science, Mountain View, CA 94035, USA*

[3]*NTT Physics and Informatics Laboratories, NTT Research, Inc., Sunnyvale, CA 94085, USA*

[4]*Kavli Institute at Cornell for Nanoscale Science, Cornell University, Ithaca, NY 14853, USA*

CONTENTS

I Simulation results 1

Supplementary Note 1. Modeling and training 2

A. SPDNNs with incoherent optical setups 2

B. SPDNNs with coherent optical setups 4

Supplementary Note 2. Classification performance 6

A. MNIST classification task 6

1. SPDNNs with incoherent optical setups 6

2. SPDNNs with coherent optical setups 9

B. CIFAR-10 classification task 12

C. Additional classification tasks 14

II Experimental setup 16

Supplementary Note 3. Incoherent optical matrix-vector multiplier 16

Supplementary Note 4. Single-photon detection by a scientific CMOS camera 18

Supplementary Note 5. Validation of the optical vector-vector multiplications 20

* sm2725@cornell.edu

† Present address: Department of Applied Physics, Yale University, New Haven, Connecticut 06511, USA

‡ pmcmahon@cornell.edu

Supplementary Note 6. Validation of the SPD activation function 21

III Implementation of SPDNNs 22

Supplementary Note 7. Adaptation to experimental limitations 22

Supplementary Note 8. Optical implementation of SPD activations 23

Supplementary Note 9. Full-optical implementation of the entire SPDNN models 31

IV Discussion 36

Supplementary Note 10. Robustness tests of SPDNNs 37

Supplementary Note 11. Noise resilience compared to conventional models 38

Supplementary Note 12. Distribution of expectation values for SPD activations 40

Supplementary Note 13. Background for stochastic computing 41

Supplementary Note 14. Prospects for future applications 42

References 44

Part I

Simulation results

In this part, we introduce the details of simulation of the single-photon-detection neural networks (SPDNNs). Each neuron activation in an SPDNN, corresponding to a readout on a single-photon detector (SPD) in an experiment, is modeled as a binary stochastic process [1–3]. For each SPD measurement, the single-shot output is either 0 or 1, with probabilities determined by the incident optical energy. The exact form of the activation function is defined by the actual physical process of single-photon detection. For an incident beam with optical energy of λ photons per detection, due to Poissonian photon statistics, the probability for an SPD to detect a click is $P_{\mathrm{SPD}}(\lambda) = 1 - e^{-\lambda}$, as shown in Figure 2 in the main text. The detected binary results are used to compute the activation values. However, due to the stochasticity and discretization in the single-photon-detection process, estimating the gradients in the loss function is challenging, and conventional backpropagation algorithms fail to train these models.

Training stochastic neuron models has been investigated for many years. One of the major families of algorithms dependent on such neurons is the Boltzmann machine [4, 5]. REINFORCE algorithms (RA) [6] update the weights in the direction of the gradients of expected reinforcement without explicitly computing them. These algorithms have been investigated and applied in different tasks to train stochastic neural networks effectively [7, 8]. In [9], many methods of estimating gradients through stochastic neurons are studied. They found that the fastest training in their experiments was achieved by the "straight-through estimator" (STE), which was previously introduced in Hinton's course, lecture 15b [10]. In our simulation of SPDNNs, we were inspired by both methods and found an estimator that trained our SPDNNs effectively, with the activation induced by the physical single-photon detection process. When using STE in a binary neural network, the binarization process, either deterministic or stochastic, is regarded as identity function during back propagation. However, if we directly use the STE to go "straight through" the entire SPD process, the training performance is not very good. This is because the STE is a biased estimator of the gradients [9, 11], meaning the expectation value of the estimator is not the same as the true expectation value of the real random variable. The biased estimation of gradients harms training accuracy over a large number of epochs. Then we looked for an unbiased estimator inspired by RA [6, 9]. We can conceptually break the single-photon detection process into two parts, a determinisic probability function P_{SPD}, and the Bernoulli sampling that introduces the stochasticity. For a Bernoulli distribution, the expectation value is the probability of 1 itself, so P_{SPD} is also the expectation value of the activation. Instead of going "straight through" the entire SPD process, we only skip the Bernoulli sampling process to avoid the uncertainty in backpropagation and include the gradients induced by the probability function to meet the expectation values of the random variable.

To enhance training effectiveness in certain cases, we introduced a slope variable, η, which modifies the intensity value within the SPD activation function: $P^{\eta}_{\mathrm{SPD}}(\lambda) = P_{\mathrm{SPD}}(\eta\lambda)$. The incorporation of a technique called "slope annealing" [12] allows controlled alteration of the gradients of the activation function, leading to more efficient navigation of the model's parameter space. Additionally, we impose an upper limit on the intensity by clamping it to a maximum value $\lambda_{\max}$. This prevents the occurrence of vanishing gradients resulting from excessively large values and the plateauing probability function. Both the application of the slope variable and intensity clamping can be utilized exclusively during the training phase. In optical implementation, the annealing factor can be absorbed in the

mapping from the trained weights to the controlled parameters on the experimental setup.

The details of the backpropagation and training process are shown in Algorithms 1 and 3, with the exact activation functions of incoherent and coherent optical setups, respectively. In the following sections, we introduce the two SPDNN setups in detail and test their performance with different tasks and architectures.

Supplementary Note 1. MODELING AND TRAINING

A. SPDNNs with incoherent optical setups

When an optical neural network (ONN) operates with incoherent light, the values of the vector elements are encoded as the intensity of light. The encoded values are non-negative, and the operations are performed by modulating the intensity of light. Thus, for an optical matrix-vector multiplier (optical MVM) operating with incoherent light, the values in an output vector z are readily the intensity to be measured by the detector, i.e., $\lambda = z$. The probability of having the SPD measurement of 1 is then $P_{\mathrm{SPD}}(\lambda(z)) = P_{\mathrm{SPD}}(z)$. This probability P_{SPD} is determined by the pre-activation value z. Thus, the SPD activation is a Bernoulli sampling of the probability P_{SPD}, $f_{\mathrm{SPD}}^{\mathrm{Incoh}}(z) = \mathbf{1}_{t<P_{\mathrm{SPD}}(z)}$, where t is a uniform random variable $t \sim U[0,1]$ and $\mathbf{1}_x$ is the indicator function on the true value of x, i.e.

$$f_{\mathrm{SPD}}^{\mathrm{Incoh}}(z) = \begin{cases} 1 & \text{with probability } p = P_{\mathrm{SPD}}(z), \\ 0 & \text{with probability } 1-p, \end{cases} \tag{1}$$

where the probability function $P_{\mathrm{SPD}}(z) = 1 - e^{-z}$. The activation in the forward propagation is calculated as $a = f_{\mathrm{SPD}}^{\mathrm{Incoh}}(z)$.

In the backward propagation, the stochastic Bernoulli sampling process is regarded as an identity function ("straight through"), so that the gradients can propagate through the whole model as it were deterministic. In this way, the SPDNNs with stochastic binary neurons can be efficiently trained. The training procedure of incoherent SPDNNs is detailed in Algorithm 1. With L layers in the neural network, the SPD activation function is applied after every layer except the output layer. In the lth layer ($l \neq L$), $z^{(l)} = a^{(l-1)}W^{(l)}$ is the direct output of the optical MVM that encodes the information of the dot product results. In an incoherent optical setup, the output values are directly encoded in light intensity, $\lambda^{(l)} = z^{(l)}$. In the training process, we clamp the intensity to a maximum value $\lambda_{\max}$ to avoid vanishing gradients with large values. Meanwhile, the clamped intensity vector $\lambda^{(l)}$ is multiplied by the slope variable η to compute the probability of detecting a click on the SPDs according to P_{SPD}: $p^{(l)} = P_{\mathrm{SPD}}(\eta\lambda^{(l)})$. Then the activation values are the Bernoulli sampling of the computed probabilities: $a^{(l)} = \mathbf{1}_{t<p^{(l)}}$, which are sent to the next layer in the forward propagation. In backward propagation, our gradient estimator assumes the gradients of the stochastic sampling process are 1: $\partial a^{(l)}/\partial p^{(l)} = 1$. Thus, during the backward pass of the lth layer, given the gradient with respect to $a^{(l)}$, $g_{a^{(l)}} = \partial C/\partial a^{(l)}$, calculated from the next layer (previous layer in backward propagation), the gradient with respect to the pre-activation $z^{(l)}$ is calculated as follows:

$$g_{z^{(l)}} = \frac{\partial a^{(l)}}{\partial z^{(l)}} \circ g_{a^{(l)}} = \frac{\partial a^{(l)}}{\partial p^{(l)}} \circ \frac{\partial p^{(l)}}{\partial \lambda^{(l)}} \circ \frac{\partial \lambda^{(l)}}{\partial z^{(l)}} \circ g_{a^{(l)}} = 1 \circ P'_{\mathrm{SPD}}(\lambda^{(l)}) \circ 1 \circ g_{a^{(l)}} = P'_{\mathrm{SPD}}(z^{(l)}) \circ g_{a^{(l)}}, \tag{2}$$

so the gradients with respect to the weights $W^{(l)}$ are $g_{W^{(l)}} = g_{z^{(l)}}^{\top} a^{(l-1)}$. In this way, the gradients can be efficiently calculated to optimize the weights using a gradient-based optimizer with a learning rate.

Algorithm 1 Physics-aware stochastic training of an SPDNN with an incoherent optical setup. N_{batch} is the batch size, N_l denotes the number of neurons in layer l and N_0 is the input size. C is the loss function. L is the number of layers. $P_{\text{SPD}}(\lambda)$ is the function of the probability to detect a click on the single-photon detector (SPD) with respect to the incident light intensity λ (in number of photons). Sample() is a probabilistic sampling of the probability. In SPDNNs, it refers to Bernoulli sampling, Sample(p) has a probability of p to be 1 and a probability of $1-p$ to be 0 (i.e. Sample(p) $\equiv \mathbf{1}_{t<p}$, $t \sim U[0,1]$). In experiments, an SPD detection intrinsically consists both of the process, the SPD activation function $f_{\text{SPD}}(\lambda) = \mathbf{1}_{t<P_{\text{SPD}}(\lambda)}$, $t \sim U[0,1]$. The λ is equivalent to the pre-activation z in an incoherent setup. Output() determines the function applied to the pre-activation right before the final output, such as Softmax or LogSoftmax. Update() specifies how to update the parameters given the calculated gradients, using optimizers such as SGD [13], Adam [14] or AdamW [15].

Require: A batch of inputs $a^{(0)}$ ($N_{\text{batch}} \times N_0$) with corresponding targets y, current weights $W^{(l)}$ ($N_l \times N_{l-1}$, $l \in \{0, 1, \dots, L\}$), current slope variable η, slope annealing factor θ, current learning rate α, decay coefficient γ and the clamped photon number $\lambda_{\max}$.
Ensure: Updated weights $W^{(l)}$ ($l \in \{0, 1, \dots, L\}$), slope η and learning rate α .

I. Forward pass
for $l = 1$ to L **do**
  $z^{(l)} \leftarrow a^{(l-1)} W^{(l)\top}$ ▷ Linear operation to compute the pre-activation values
  $\lambda^{(l)} \leftarrow z^{(l)}$ ▷ For incoherent light, intensity is directly modulated
  $\lambda^{(l)} \leftarrow \min(\lambda^{(l)}, \lambda_{\max})$ ▷ Clamp the maximum intensity
  if $l < L$ **then**
    $p^{(l)} \leftarrow P_{\text{SPD}}(\eta \cdot \lambda^{(l)})$ ▷ The probability of detecting a click, with the slope η applied
    $a^{(l)} \leftarrow \text{Sample}(p^{(l)})$ ▷ SPD activation values
  end if
end for
$a^{(L)} \leftarrow \text{Output}(\lambda^{(L)})$ ▷ Final output function
II. Backward pass
Compute $g_{a^{(L)}} = \frac{\partial C}{\partial a^{(L)}}$ knowing $a^{(L)}$ and y.
$g_{z^{(L)}} \leftarrow \frac{\partial a^{(L)}}{\partial z^{(L)}} \circ g_{a^{(L)}}$
for $l = L$ to 1 **do**
  if $l < L$ **then**
    $g_{p^{(l)}} \leftarrow g_{a^{(l)}}$ ▷ "Straight-through" here, skip the Bernoulli process
    $g_{z^{(l)}} \leftarrow P'_{\text{SPD}}(z^{(l)}) \circ g_{p^{(l)}}$ ▷ $\frac{\partial p^{(l)}}{\partial z^{(l)}} = \frac{\partial p^{(l)}}{\partial \lambda^{(l)}} \circ \frac{\partial \lambda^{(l)}}{\partial z^{(l)}} = P'_{\text{SPD}}(\lambda^{(l)}) \circ 1 = P'_{\text{SPD}}(z^{(l)})$
  end if
  $g_{a^{(l-1)}} \leftarrow g_{z^{(l)}} W^{(l)}$
  $g_{W^{(l)}} \leftarrow g_{z^{(l)}}^{\top} a^{(l-1)}$ ▷ The gradients for $W^{(l)}$
end for
III. Parameter update
for $l = 1$ to L **do**
  $W^{(l)} \leftarrow \text{Update}(W^{(l)}, g_{W^{(l)}}, \alpha)$ ▷ Update the weights
  $W^{(l)} \leftarrow \max(W^{(l)}, 0)$ ▷ Clip the weights to be non-negative for the incoherent setup
end for
$\eta \leftarrow \theta\eta$ ▷ Update the slope
$\alpha \leftarrow \gamma\alpha$ ▷ Update the learning rate

Note that for an incoherent optical setup, the elements in the weights (realized by intensity modulations) are also non-negative, so the updated weights need to be clamped to non-negative values after each optimization step. After each optimization step, the slope variable is updated by multiplying by a factor θ, as the "slope annealing" trick [12] to improve the training performance when necessary.

During the inference of a trained model, the forward pass of test inputs is similar to the training process, except

that the maximum clamping $\lambda_{\max}$ is not applied. Additionally, to control the level of uncertainty in the stochastic neural networks, we can choose to use multiple shots of SPD measurements during each inference. In a "K-shot" inference, we use K shots of binary SPD readouts, and the final activation value of the neuron, denoted as $a^{[K]}$, is the average of the K independent stochastic binary values. This process essentially involves integrating a few more photons using the SPD, as is usually done for conventional ONN implementation [16].

For a single shot of SPD measurement per activation, $a^{[1]} = a \in \{0, 1\}$, while for K shots, $a^{[K]} = \frac{1}{K}\sum_{k=1}^{K} a_k \in \{0, 1/K, 2/K, \ldots, 1\}$. This approach reduces the uncertainty in the models, resulting in more precise output values. In the ideal case where an infinite number of shots are integrated ($K \to \infty$), the activation $a^{[\infty]}$ would converge to the expectation value without stochasticity, denoted as $a^{[\infty]} = \mathbb{E}[a] = P_{\text{SPD}}(z)$. As we will see in Supplementary Note 2 A 1, the SPDNN models have higher test accuracy as the shots per activation K increases. The detailed inference procedure is explained in Algorithm 2.

Algorithm 2 Inference of an SPDNN with an incoherent optical setup. L is the number of layers. N_{batch} is the batch size, N_l denotes the number of neurons in layer l and N_0 is the input size. K is the number of shots used in one inference. Sampling(), experiment. The predictions of an inference is based on the label of the output node with the maximum output value.

Require: A batch of test inputs $a^{(0)}$ ($N_{\text{batch}} \times N_0$) and trained weights $W^{(l)}$ ($N_l \times N_{l-1}$, $l \in \{0, 1, \ldots, L\}$), slope annealing factor η.
Ensure: The output $a^{(L)}$.

for $l = 1$ to L **do**
  $z^{(l)} \leftarrow a^{(l-1)} W^{(l)\top}$ ▷ Linear operation to compute the pre-activation
  $\lambda^{(l)} \leftarrow z^{(l)}$ ▷ For incoherent light, intensity is directly modulated
  if $l < L$ **then** ▷ SPD activation process
    $p^{(l)} \leftarrow P_{\text{SPD}}(\eta \cdot \lambda^{(l)})$ ▷ The probability of detecting a click, with the slope η applied
    for $k = 1$ to K **do** ▷ K shots in one inference
      $a^{(l),k} \leftarrow \text{Sampling}(p^{(l)})$ ▷ SPD output for each shot
    end for
    $a^{(l)} \leftarrow \frac{1}{K}\sum_{k=1}^{K} a^{(l),k}$ ▷ Average over all K shots for the activation values
  end if
end for
$a^{(L)} \leftarrow \lambda^{(L)}$ ▷ Use the output intensity directly in the inference

B. SPDNNs with coherent optical setups

In coherent optical MVMs [17–23], the information is conveyed through both the amplitude and phase of light states. These multipliers have the potential to encode complex numbers using arbitrary phase, but in most applications, only phases of 0 and π are used for positive and negative real-number values, to align with conventional machine learning models. Our work focuses on real-valued coherent optical MVMs. Now that the information is encoded in the amplitude and phase instead of the intensity, the photon detection process involves measuring the square modulus of the complex number, which adds an extra square function to the pre-activation values. Thus, the coherent SPD activation function is $f_{\text{SPD}}^{\text{Coh}}(z) = \mathbf{1}_{t < P_{\text{SPD}}(z^2)}$, where t is a uniform random variable $t \sim U[0, 1]$ and $\mathbf{1}_x$ is the indicator

function on the true value of x, i.e.

$$f_{\text{SPD}}^{\text{Coh}}(z) = \begin{cases} 1 & \text{with probability } p = P_{\text{SPD}}(z^2), \\ 0 & \text{with probability } 1-p, \end{cases} \tag{3}$$

where $P_{\text{SPD}}(z^2) = 1 - e^{-z^2}$. The activation in the forward propagation is calculated by $a = f_{\text{SPD}}^{\text{Coh}}(z)$. The expectation of the coherent SPD activation is $\mathbb{E}[f_{\text{SPD}}^{\text{Coh}}] = P_{\text{SPD}}(z^2)$.

Algorithm 3 Physics-aware stochastic training of an SPDNN with coherent light. N_{batch} is the batch size, N_l denotes the number of neurons in layer l and N_0 is the input size. C is the loss function. L is the number of layers. $P_{\text{SPD}}(\lambda)$ is the function of the probability to detect a click on the single-photon detector (SPD) with respect to the incident light intensity λ (in number of photons). Sample() is a probabilistic sampling of the probability. In SPDNNs, it refers to Bernoulli sampling, Sample(p) has a probability of p to be 1 and a probability of $1-p$ to be 0 (i.e. Sample(p) $\equiv \mathbf{1}_{t<p}$, $t \sim U[0,1]$). For a coherent setup, $\lambda = z^2$ where z is the pre-activation, output of a matrix-vector multiplier. Output() determines the function applied to the pre-activation right before the final output, such as Softmax or LogSoftmax. Update() specifies how to update the parameters given the calculated gradients, using optimizers such as SGD [13], Adam [14] or AdamW [15].

Require: A batch of inputs $a^{(0)}$ ($N_{\text{batch}} \times N_0$) with corresponding targets y, current weights $W^{(l)}$ ($N_l \times N_{l-1}$, $l \in \{0, 1, \ldots, L\}$), current slope variable η, slope annealing factor θ, current learning rate α, decay coefficient γ and the clamped photon number $\lambda_{\max}$.

Ensure: Updated weights $W^{(l)}$ ($l \in \{0, 1, \ldots, L\}$), slope η and learning rate α.

I. Forward pass
for $l = 1$ to L **do**
  $z^{(l)} \leftarrow a^{(l-1)} W^{(l)\top}$ ▷ Linear operation to compute the pre-activation
  $\lambda^{(l)} \leftarrow (z^{(l)})^2$ ▷ For coherent light, intensity is the square of the amplitude
  $\lambda^{(l)} \leftarrow \min(\lambda^{(l)}, \lambda_{\max})$ ▷ Clamp the maximum intensity
  if $l < L$ **then**
    $p^{(l)} \leftarrow P_{\text{SPD}}(\lambda^{(l)})$ ▷ The probability of detecting a click on the SPDs
    $a^{(l)} \leftarrow \text{Sample}(p^{(l)})$ ▷ SPD output for each shot
  end if
end for
$a^{(L)} \leftarrow \text{Output}(\lambda^{(L)})$ ▷ Final output function
II. Backward pass
Compute $g_{a^{(L)}} = \frac{\partial C}{\partial a^{(L)}}$ knowing $a^{(L)}$ and y.
$g_{z^{(L)}} \leftarrow \frac{\partial a^{(L)}}{\partial z^{(L)}} \circ g_{a^{(L)}}$
for $l = L$ to 1 **do**
  if $l < L$ **then**
    $g_{p^{(l)}} \leftarrow g_{a^{(l)}}$ ▷ "Straight-through" here, skip the Bernoulli process
    $g_{z^{(l)}} \leftarrow 2z^{(l)} \circ P'_{\text{SPD}}\left((z^{(l)})^2\right) \circ g_{p^{(l)}}$ ▷ $\frac{\partial p^{(l)}}{\partial z^{(l)}} = \frac{\partial p^{(l)}}{\partial \lambda^{(l)}} \circ \frac{\partial \lambda^{(l)}}{\partial z^{(l)}} = 2z^{(l)} \circ P'_{\text{SPD}}\left((z^{(l)})^2\right)$
  end if
  $g_{a^{(l-1)}} \leftarrow g_{z^{(l)}} W^{(l)}$
  $g_{W^{(l)}} \leftarrow g_{z^{(l)}}^{\top} a^{(l-1)}$ ▷ The gradients with respect to $W^{(l)}$
end for
III. Parameter update
for $l = 1$ to L **do**
  $W^{(l)} \leftarrow \text{Update}(W^{(l)}, g_{W^{(l)}}, \alpha)$ ▷ Update the weights
end for
$\eta \leftarrow \theta\eta$ ▷ Update the slope
$\alpha \leftarrow \gamma\alpha$ ▷ Update the learning rate

The coherent activation function, depicted in Figure 4a of the main text, exhibits a distinct "V" shape due to the additional square operation, which is symmetric about the y axis. It could be problematic as an activation function

[24]. One possible solution is to modify the information encoding and detection scheme to alter the exact form of $\lambda(z)$ (e.g. [21]). However, in this section, we have chosen to employ the most straightforward intensity-detection scenario, which does not necessitate modifications to conventional ONN implementation. Remarkably, despite its simplicity, this activation function delivers comparable performance and demonstrates impressive results. By adopting this approach, we alleviate experimental complexities while ensuring reliable inference in our SPDNN models.

Supplementary Note 2. CLASSIFICATION PERFORMANCE

A. MNIST classification task

To illustrate the capability of the SPD activation function, we first use it in a simple multi-layer perceptron (MLP) architecture and train the models on the benchmark MNIST classification task. The models have a structure of $784 \to N \to 10$ with N neurons in the hidden layer, as discussed in the main text. The MNIST dataset has 60,000 images for training and 10,000 images for testing. Each image is grayscale and has $28 \times 28 = 784$ pixels. To meet the non-negative encoding of incoherent light, the input images are normalized to have pixel values ranging from 0 to 1.

1. SPDNNs with incoherent optical setups

The models consist of two linear layers: the $784 \to N$ hidden layer has the weight matrix $W^{(1)}$ with a shape of $N \times 784$, and the $N \to 10$ output layer has the weight matrix $W^{(2)}$ with a shape of $10 \times N$. The SPD activation function is applied to each hidden neuron after the linear operation of $W^{(1)}$ to compute the neuron activations; then the computed activation values are passed to the output layer to produce the output vectors. The elements in the first linear operation, $W^{(1)}$, are clamped to be non-negative to meet the requirement of an incoherent optical setup. In general, real-valued weights can be realized with an incoherent optical MVM if some digital-electronic post-processing is available. In our case, where the activations are measured by SPDs, the activation function is directly applied in the single-photon detection process, which makes digital post-processing impossible. Similarly, biases of the linear operations are also disabled. If we want to get away with digital post-processing by applying a bias term directly to the optical intensity, at the level of a few photons, the approach is also challenging in experiments. However, since the output layer is implemented using conventional optical computing with a higher signal-to-noise ratio (SNR), we can effectively implement real-valued weights of $W^{(2)}$. In optical implementation, this would involve extra operations to map these values onto the incoherent setup.

During the training process, we apply the LogSoftmax function to the output vectors and use the cross-entropy loss to construct the loss function. To avoid the issue of vanishing gradients, we clamp the pre-activation values at $\lambda_{\max} = 3$ photons. It is important to note that due to the stochastic nature of the neural networks, each forward pass generates different output values, even with the same weights and inputs. However, we only use a single forward pass in each training epoch, which has been shown to be the most efficient training approach. The stochasticity introduced in each forward propagation could add to the random search of the stochastic optimizer itself, helping with the training process.

We have found that using the SGD optimizer [13] with small learning rates leads to better accuracy compared

to other optimizers, such as AdamW [15]. Although training with SGD takes longer overall, it helps us achieve a better-optimized model in the end. For our final results, we used a batch size of 128 and a learning rates of 0.001 for the hidden layer and 0.01 for the output layer in the SGD optimizer. We trained each SPDNN model for 10,000 epochs to obtain optimized parameters, and an even higher number of epochs may be needed to achieve better accuracy. Given the small learning rate and the significant amount of noise in the model, the number of epochs required is much larger than what is typically seen in common neural network training processes. The training and test errors for an incoherent MLP SPDNN with a structure of $784 \to 400 \to 10$ are shown in Supplementary Figure 1. The training process was performed on a GPU (Tesla V100-PCIE-32GB) and took approximately eight hours to complete.

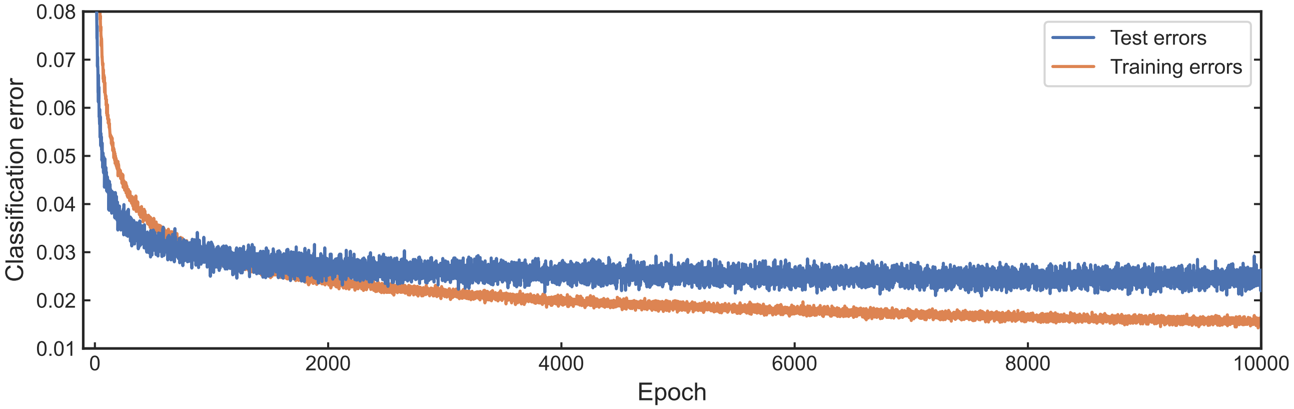

Supplementary Figure 1. Training curves of an incoherent SPDNN model for MNIST classification. The plot illustrates the progression of test and training errors throughout the training process of an incoherent SPDNN model with an MLP architecture of $784 \to 400 \to 10$. The optimization is conducted using an SGD optimizer with learning rates of 0.001 for the hidden layer and 0.01 for the output layer. The final trained model is obtained at 10,000 epochs.

The magnitude of the weight element values in the first linear layer, $W^{(1)}$, is influenced by the range of values in the input vectors, $a^{(0)}$, and the specific form of the SPD activation function, $f_{\mathrm{SPD}}^{\mathrm{Incoh}}(z)$. In the forward pass of an incoherent SPDNN, the pre-activation values, $z^{(1)}$, are computed as $z^{(1)} = a^{(0)} W^{(1)\top}$. The activation function, $f_{\mathrm{SPD}}^{\mathrm{Incoh}}(z)$, is defined as $\mathbf{1}_{t<P_{\mathrm{SPD}}(z)}$, where t is a random variable uniformly distributed between 0 and 1, and $P_{\mathrm{SPD}}(z)$ represents the probability of photon detection. When the input vectors, $a^{(0)}$, are normalized to the range of 0 to 1, the weight elements in $W^{(1)}$ are optimized based on the specific form of P_{SPD} because it depends on the exact value of pre-activations z. In our simulation of an incoherent SPDNN, the elements of $z^{(1)}$ are represented in terms of photon numbers, where the value 1 corresponds to 1 photon. When $z \gtrsim 3$, P_{SPD} reaches the plateau part of the probability function. Thus, we have to make sure the value of the pre-activation to be around 1 photon to ensure an effective forward pass. When considering a uniform bright image where each element has the maximum value of 1, and with an input vector size of $28 \times 28 = 784$, if we aim for an output value of approximately 1 photon, the average value of the weight elements in $W^{(1)}$ should be around $1/784 \approx 0.0013$. The average pixel value in the MNIST dataset is approximately 0.13 (when each pixel value is normalized to the range of 0 to 1). Based on this, we can estimate that to achieve an output value of approximately 1 photon, the average weight element value should be around 0.01. Taking into account that both the input images and weight matrices tend to be sparse, this estimation may be slightly lower than the actual scenario. Supplementary Figure 2 illustrates the matrix elements of $W^{(1)}$ for a model with $N = 100$

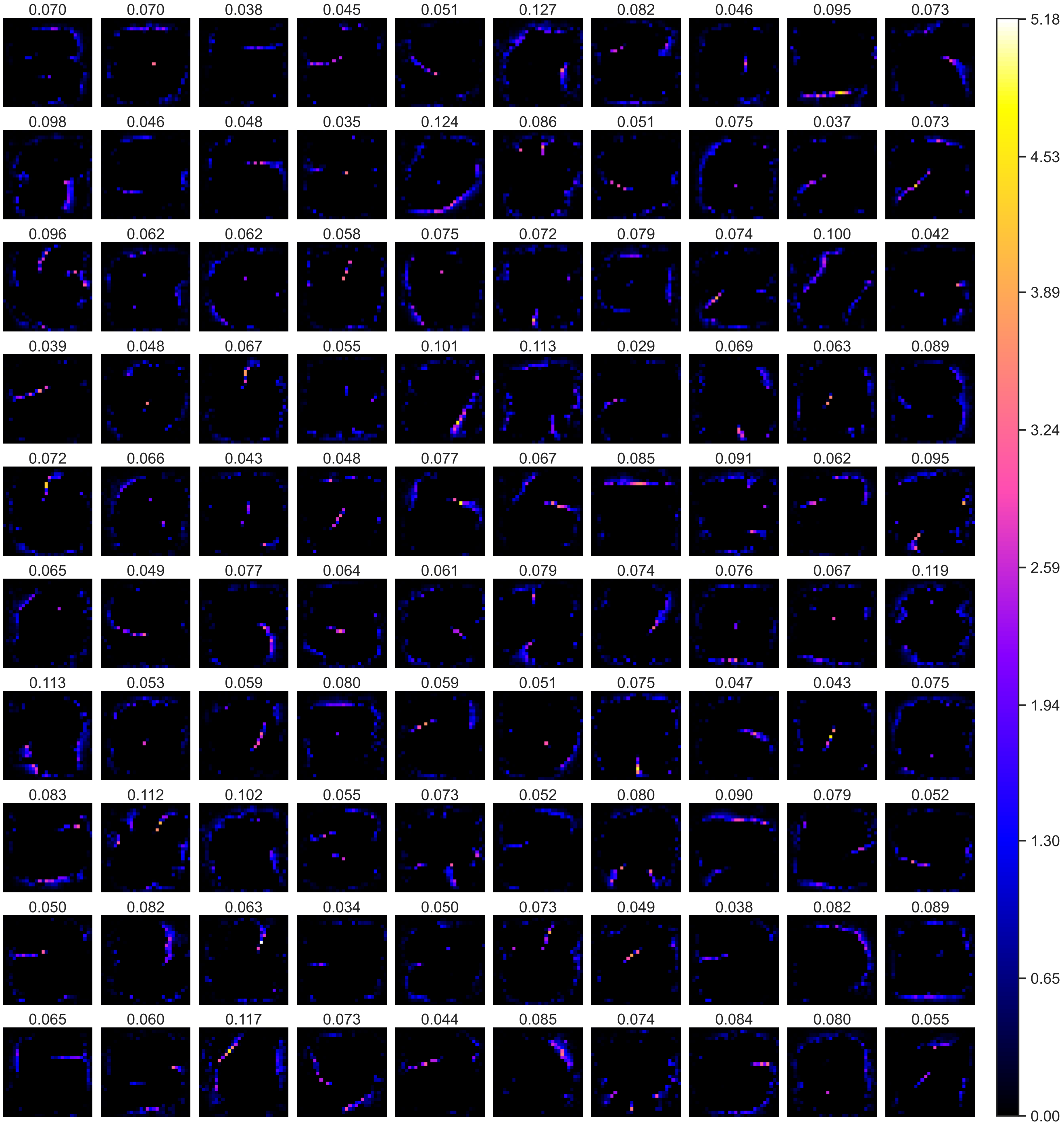

Supplementary Figure 2. Visualization of weight elements in the first linear layer of an incoherent SPDNN. The architecture of this model is $784 \to 100 \to 10$, and we display the weight matrix $W^{(1)}$ of the first layer (with dimensions 100×784). Each block represents a row vector in $W^{(1)}$ containing 784 elements. These column vectors are rearranged to form a 2D block with dimensions 28×28, matching the original shape of the MNIST input images. The 100 rows in $W^{(1)}$, corresponding to the 100 hidden neurons in the neural network, are arranged in a 10×10 grid to be visualized. The average value of each block is indicated at the top, and the overall average value of the weight matrix is approximately ~ 0.07.

hidden neurons. The weight elements range from 0 to 5.18, with an average value of 0.07. Each block represents a row vector of size 784, rearranged in the form of 28×28. The average value of $W^{(1)}$ may vary slightly in different network structures, ranging from 0.06 to 0.08.

During the inference of SPDNNs, the pixel values of the test images are normalized to the range of 0 to 1 as well. This will be correspond to the dynamic range on the optical setup. We trained incoherent MLP-SPDNN models with varying numbers of hidden neurons, N, ranging from 10 to 400. As discussed in Section Supplementary Note 1 A, we can adjust the number of SPD measurements per activation, denoted as K, to control the level of stochasticity in the models.

Model	$K = 1$	$K = 2$	$K = 3$	$K = 5$	$K = 7$	$K = 10$	$K \to \infty$
784–10–10	78.03 ± 0.32	83.18 ± 0.26	84.79 ± 0.22	86.13 ± 0.17	86.65 ± 0.17	87.08 ± 0.16	87.91 ± 0.00
784–20–10	86.74 ± 0.24	89.98 ± 0.18	90.96 ± 0.15	91.71 ± 0.13	92.00 ± 0.13	92.22 ± 0.13	92.66 ± 0.00
784–50–10	93.04 ± 0.16	94.49 ± 0.15	94.92 ± 0.12	95.24 ± 0.11	95.38 ± 0.10	95.47 ± 0.09	95.73 ± 0.00
784–100–10	95.20 ± 0.16	96.24 ± 0.11	96.53 ± 0.10	96.75 ± 0.09	96.85 ± 0.07	96.91 ± 0.07	97.02 ± 0.00
784–200–10	96.62 ± 0.12	97.33 ± 0.10	97.54 ± 0.08	97.70 ± 0.08	97.75 ± 0.08	97.80 ± 0.06	97.98 ± 0.00
784–300–10	97.00 ± 0.12	97.61 ± 0.08	97.80 ± 0.08	97.93 ± 0.07	97.97 ± 0.06	98.01 ± 0.05	98.12 ± 0.00
784–400–10	97.31 ± 0.11	97.85 ± 0.10	98.01 ± 0.09	98.15 ± 0.06	98.20 ± 0.06	98.27 ± 0.05	98.41 ± 0.00

Supplementary Table 1. Test accuracy (%) of incoherent MLP-SPDNNs on MNIST with varying hidden layer size N and shots per activation K. These models have an MLP structure of $784 \to N \to 10$, where N represents the number of hidden neurons. Each hidden neuron uses K shots of binary SPD readouts to compute its activation value. The reported test accuracy values are obtained by calculating the mean value and standard deviation over 100 repetitions of inferences on the MNIST test set, which comprises 10,000 images.

The results of the MNIST test accuracy for different combinations of N and K are summarized in Supplementary Table 1. The values of N include 10, 20, 50, 100, 200, 300, and 400, while K takes on the values of 1, 2, 3, 5, 7, 10, and ∞. In the case of $K \to \infty$, we use the expectation of the activation values, P_{SPD}, as the activation function, which is equivalent to integrating an infinite number of shots per SPD detection. This serves as an upper bound that is approached as K increases. Due to the stochastic nature of SPDNNs, the output vectors vary across different repetitions of inference. To capture the overall behavior of the models, we repeated the full inference process 100 times for each structure with N hidden neurons and K shots per activation. This allows us to calculate the mean test accuracy and standard deviation, representing the distribution of test accuracies. Each independent repetition of inference uses the MNIST test dataset, consisting of 10,000 images. We observe that as either N or K increases, the mean test accuracy tends to improve while the standard deviation decreases.

2. SPDNNs with coherent optical setups

The MNIST handwritten-digit classification task was performed using the same simulation configurations as with incoherent SPDNNs, but but using the coherent SPD activation function and real-number operations. Unlike the previous case, no clamping of the weights was necessary. The models were trained using the SGD optimizer with a learning rate of 0.01 for the hidden layers and 0.001 for the last linear layer, for a period of 10,000 epochs. To evaluate the impact of model size, we trained models with both one and two hidden layers. The training curves of the model with the structure of $784 \to 400 \to 400 \to 10$ are shown in Supplementary Figure 3. The results for models with different structures and shots of SPD measurements per activation can be found in Supplementary Table 2, and the

weights of a model with the structure of 784 → 100 → 10 are illustrated in Supplementary Figure 4.

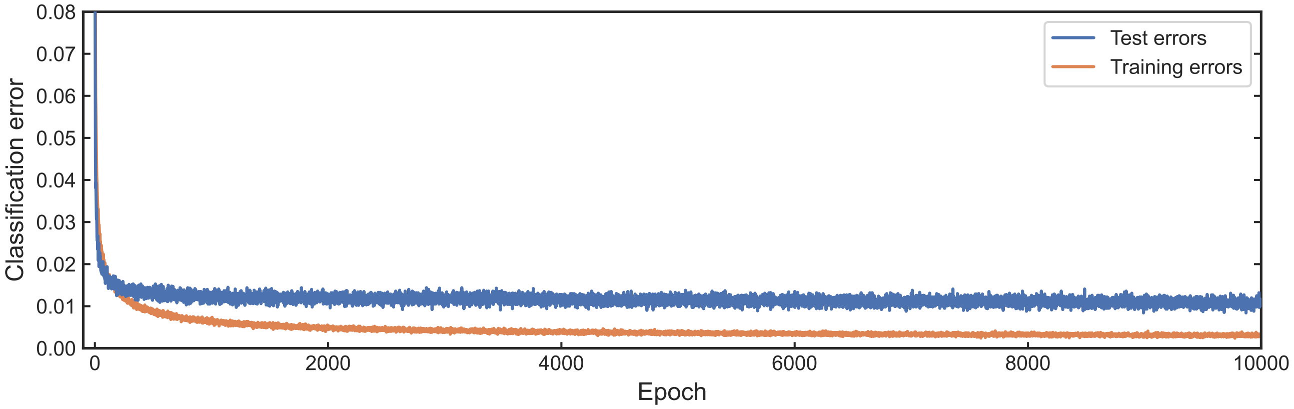

Supplementary Figure 3. Training curves of a coherent SPDNN model on MNIST classification. The plot shows the evolution of the test and training errors during the training process of a coherent SPDNN model with an MLP architecture of 784 → 400 → 400 → 10 neurons. The optimization is performed using SGD with different learning rates for the hidden (0.001) and output (0.01) layers. The end result of the training is represented by the final values of the 10,000 epochs.

Model	$K = 1$	$K = 2$	$K = 3$	$K = 5$	$K = 7$	$K = 10$	$K \to \infty$
784–10–10	80.21 ± 0.27	85.63 ± 0.24	87.49 ± 0.19	88.85 ± 0.15	89.42 ± 0.15	89.87 ± 0.14	90.70 ± 0.00
784–20–10	89.31 ± 0.23	92.43 ± 0.17	93.42 ± 0.14	94.10 ± 0.13	94.39 ± 0.14	94.60 ± 0.11	95.05 ± 0.00
784–25–10	91.51 ± 0.20	94.16 ± 0.16	94.89 ± 0.14	95.47 ± 0.12	95.70 ± 0.10	95.86 ± 0.08	96.10 ± 0.00
784–30–10	92.72 ± 0.19	94.92 ± 0.13	95.58 ± 0.12	96.05 ± 0.10	96.26 ± 0.09	96.41 ± 0.08	96.74 ± 0.00
784–50–10	95.50 ± 0.15	96.88 ± 0.11	97.26 ± 0.09	97.55 ± 0.08	97.67 ± 0.07	97.77 ± 0.07	97.93 ± 0.00
784–100–10	97.41 ± 0.13	98.16 ± 0.08	98.36 ± 0.08	98.52 ± 0.07	98.58 ± 0.06	98.61 ± 0.05	98.70 ± 0.00
784–200–10	98.34 ± 0.10	98.76 ± 0.07	98.88 ± 0.07	98.97 ± 0.05	99.00 ± 0.05	99.04 ± 0.04	99.12 ± 0.00
784–400–10	98.64 ± 0.08	98.95 ± 0.06	99.04 ± 0.05	99.09 ± 0.05	99.12 ± 0.04	99.14 ± 0.03	99.19 ± 0.00
784–400–400–10	98.95 ± 0.08	99.21 ± 0.05	99.29 ± 0.04	99.33 ± 0.04	99.35 ± 0.04	99.37 ± 0.03	99.40 ± 0.00
784–C16–400–10	99.33 ± 0.06	99.45 ± 0.04	99.47 ± 0.04	99.50 ± 0.04	99.51 ± 0.03	99.52 ± 0.03	99.54 ± 0.00

Supplementary Table 2. Test accuracy (%) of coherent SPDNN models on MNIST with different model structures and shots per activation K. These models have an MLP structure with one or two hidden layers with the number of neurons denoted in the table, except for the last model, which has a convolutional layer denoted as "C16" with 16 output channels and followed by a 2×2 average pooling and the following linear layers. The mean accuracy and standard deviation are calculated based on 100 repetitions of inferences using the MNIST test set of 10,000 images.

Furthermore, convolutional SPDNNs were also used for MNIST classification. The architecture included a convolutional layer with 16 output channels, a kernel size of 5×5 and a stride of 1. An SPD activation function was immediately applied after each convolution layer, without batch normalization. Average pooling of 2×2 was performed after each of the SPD activations. After the convolution layer, the total number of features was 3136, then the convolution layers were followed by a linear model of 3136 → 400 → 10, with the SPD activation function applied at each of the 400 hidden neurons as well. This structure is depicted in Figure 4b in the main text. For optimization, we used an SGD optimizer with a learning rate of 0.01 for the entire model. The convolutional SPDNN model can be optimized easily without fine-tuning the parameters. After 200 epochs, the accuracy quickly reached 99.4%.

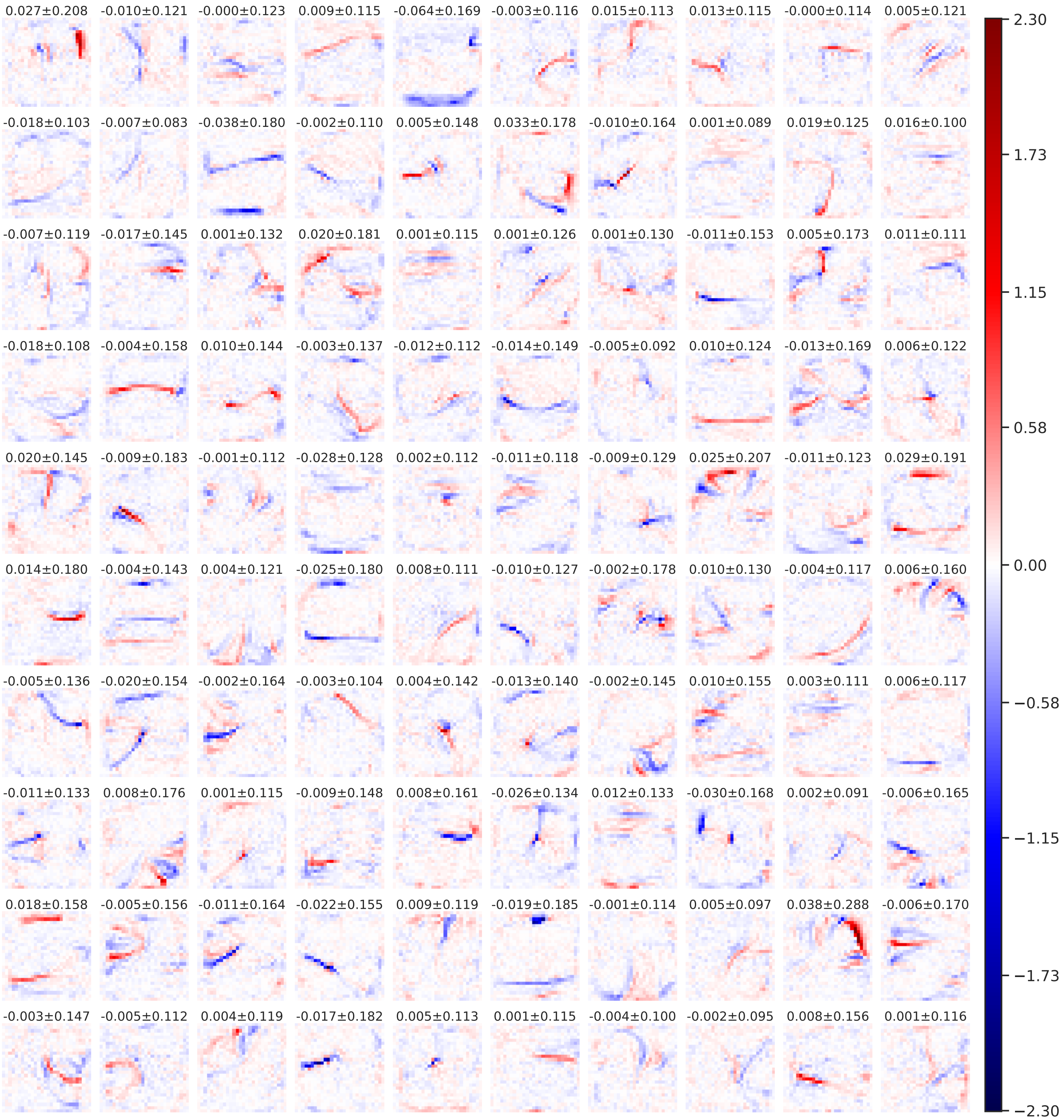

Supplementary Figure 4. Visualization of weight elements in the first linear layer of a coherent SPDNN. The architecture of this model is $784 \to 100 \to 10$, and we display the weight matrix $W^{(1)}$ of the first layer (with dimensions 100×784). Each block represents a row vector in $W^{(1)}$ containing 784 elements. These column vectors are rearranged to form a 2D block with dimensions 28×28, matching the original shape of the MNIST input images. The 100 rows in $W^{(1)}$, corresponding to the 100 hidden neurons in the neural network, are arranged in a 10×10 grid to be visualized. The average value and standard deviation of the elements in each block are indicated at the top.

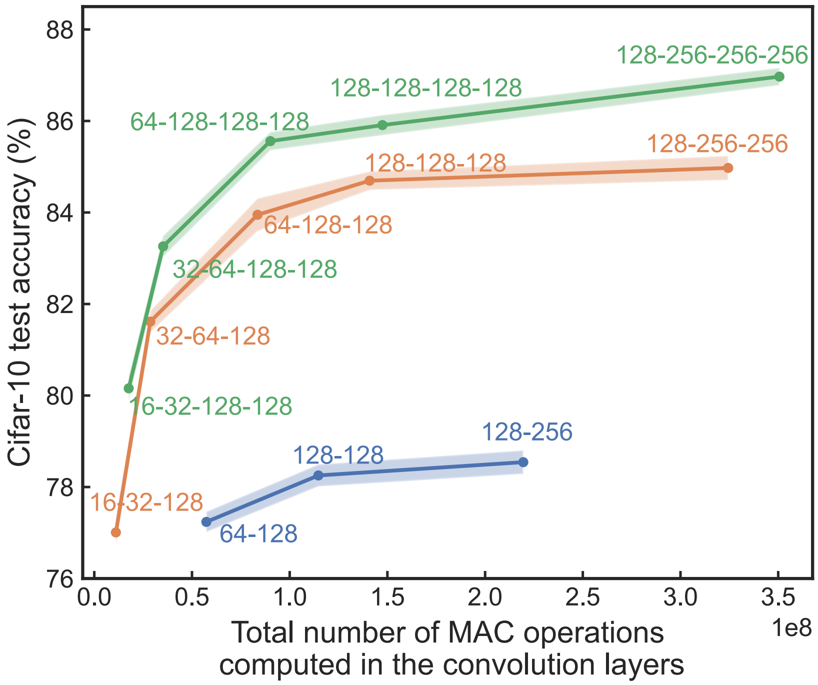

Supplementary Figure 5. Test accuracy of convolutional SPDNN models on CIFAR-10 classification. The plot shows the mean test accuracy (data points) and the corresponding standard deviation (shaded region), calculated by averaging the results of 100 repeated inference runs. The number of output channels in the convolutional layers is indicated near each data point, with different colors denoting different numbers of convolutional layers. The linear layers following the convolutional layers have a consistent structure of $400 \to 10$, with SPD activation applied to the 400 neurons.

B. CIFAR-10 classification task

The CIFAR-10 dataset [25] has 60,000 images, each having $3 \times 32 \times 32$ pixels with 3 color channels, that belong to 10 different categories, representing airplanes, automobiles, birds, cats, deer, dogs, frogs, horses, ships and trucks. The dataset is partitioned into a training set with 50,000 images and a test set with 10,000 images. The pixel values have been normalized using the mean value of $(0.4914, 0.4822, 0.4465)$ and standard deviation of $(0.2471, 0.2435, 0.2616)$ for each of the color channels. To boost performance, data augmentation techniques including random horizontal flips (50% probability) and random 32×32 crops (with 4-pixel padding) were implemented during training. We used the AdamW optimizer [15] with a learning rate of 0.0005 and betas of $(0.99, 0.98)$. The models were trained for thousands of epochs.

The convolutional SPDNNs have a structure where the SPD activation function is applied after each convolution layer and before an average pooling of 2×2. The final architecture consists of a series of convolution layers followed by a linear layer of 400 neurons, and a final layer of $400 \to 10$ for the output. Similar to the convolutional models trained for MNIST, the convolutional layers use a kernel size of 5×5, a stride size of 1 and padding of 2. Batch

normalization was used in the models after each convolutional layer. Either SPD or ReLU activation function was applied to each of the 400 neurons in the first linear layer, as depicted in Figure 4e in the main text.

After N_{conv} convolutional layers ($N_{\text{conv}} = 2$, 3 or 4 in this case) with the number of output channels of the last one to be $N_{\text{chan}}^{\text{last}}$ (either 128 or 256 in this case), the feature map of $(32/2^{N_{\text{conv}}})^2 \times N_{\text{chan}}^{\text{last}}$ is flattened to a vector, followed by two linear layers of $(32/2^{N_{\text{conv}}})^2 N_{\text{chan}}^{\text{last}} \to 400 \to 10$. In addition to the results presented in Figure 4e in the main text, we experimented with more architectures ranging from 2 to 4 convolution layers, and the results are displayed in Supplementary Figure 5. In these models, only the SPD activation function was used. The x-axis of the plot represents the number of multiply–accumulate (MAC) operations in the convolutional layers. The layout of the number of channels for each convolution layer is noted around each data point for each model. For example, "64–128" indicates that there are two convolution layers each with 64 and 128 output channels, respectively. These mean values (data points) and standard deviations (shaded area) of the test accuracies are obtained from 100 repeated inference, and the activations only involved a single shot of SPD measurement ($K = 1$) in all the neurons, including the convolutional and linear layers.

We further investigated the effects of multiple shots of SPD measurements per activation in the SPD activations in convolutional (K_{conv}) and linear (K_{lin}) layers, respectively. We chose to test a model with four convolutional layers of 128, 256, 256, and 256 output channels and varied the K_{lin} and K_{conv} to see the test accuracies. The results are summarized in Supplementary Table 3.

	$K_{\text{lin}} = 1$	$K_{\text{lin}} = 2$	$K_{\text{lin}} = 3$	$K_{\text{lin}} = 5$	$K_{\text{lin}} = 10$	$K_{\text{lin}} \to \infty$
$K_{\text{conv}} = 1$	86.94 ± 0.23	87.11 ± 0.21	87.17 ± 0.21	87.23 ± 0.19	87.26 ± 0.20	87.28 ± 0.21
$K_{\text{conv}} = 2$	88.65 ± 0.18	88.77 ± 0.18	88.83 ± 0.18	88.88 ± 0.16	88.86 ± 0.18	88.90 ± 0.16
$K_{\text{conv}} = 3$	89.16 ± 0.15	89.26 ± 0.15	89.31 ± 0.16	89.33 ± 0.15	89.35 ± 0.14	89.39 ± 0.14
$K_{\text{conv}} = 5$	89.55 ± 0.14	89.68 ± 0.15	89.71 ± 0.14	89.74 ± 0.13	89.73 ± 0.13	89.77 ± 0.13
$K_{\text{conv}} = 10$	89.82 ± 0.12	89.91 ± 0.11	89.95 ± 0.10	90.00 ± 0.11	90.00 ± 0.12	90.02 ± 0.13
$K_{\text{conv}} \to \infty$	90.09 ± 0.09	90.20 ± 0.08	90.21 ± 0.07	90.25 ± 0.07	90.26 ± 0.05	90.31 ± 0.00

Supplementary Table 3. Test accuracy (%) of the convolutional SPDNN on CIFAR-10 with varying shots per activation K in the convolutional and linear layers. The SPDNN model in this table consists of four convolutional layers with 128, 256, 256, and 256 output channels, respectively. The convolutional layers are followed by a linear layer with 400 neurons and an output layer with 10 neurons. The SPD activation function is applied to each of the 400 neurons in the first linear layer. K_{conv} represents the number of shots of SPD readouts per activation in the convolutional layers, while K_{lin} represents the shots per activation in the linear layer. The mean accuracy and standard deviation are calculated based on 100 repetitions of inferences using the CIFAR-10 test set of 10,000 images.

In these SPDNNs, the number of operations in the output layer is negligible compared to the entire models. In terms of the number of MAC operations (dot products, DPs), $N_{\text{MAC}}^{\text{out}} = 4000$ ($N_{\text{DP}}^{\text{out}} = 10$). The number of dot products, or the activation size, is directly related to the number of optical detections in ONN implementations. The output layer is the only layer that needs to be implemented with a "high SNR" (see Figure 1 in the main text). The small portion of operations in this layer indicates the capability of low-SNR stochastic layers in a deeper model. This further suggests the potential to leverage stochastic physical systems with low SNRs to perform reliable neural-network inference.

Model	$N_{\text{MAC}}^{\text{total}}$	$N_{\text{MAC}}^{\text{out}}/N_{\text{MAC}}^{\text{total}}$	$N_{\text{DP}}^{\text{total}}$	$N_{\text{DP}}^{\text{out}}/N_{\text{DP}}^{\text{total}}$
C64–C128	5.73×10^{7}	6.60×10^{-5}	2.29×10^{6}	4.36×10^{-6}
C128–C128	1.15×10^{8}	3.40×10^{-5}	4.59×10^{6}	2.18×10^{-6}
C128–C256	2.20×10^{8}	1.80×10^{-5}	8.78×10^{6}	1.14×10^{-6}
C16–C32–C128	1.11×10^{7}	3.37×10^{-4}	4.42×10^{5}	2.26×10^{-5}
C32–C64–C128	2.87×10^{7}	1.36×10^{-4}	1.15×10^{6}	8.72×10^{-6}
C64–C128–C128	8.36×10^{7}	4.70×10^{-5}	3.34×10^{6}	2.99×10^{-6}
C128–C128–C128	1.41×10^{8}	2.80×10^{-5}	5.64×10^{6}	1.77×10^{-6}
C128–C256–C256	3.24×10^{8}	1.20×10^{-5}	1.30×10^{7}	7.71×10^{-7}
C16–C32–C128–C128	1.76×10^{7}	2.24×10^{-4}	7.05×10^{5}	1.42×10^{-5}
C32–C64–C128–C128	3.52×10^{7}	1.13×10^{-4}	1.41×10^{6}	7.10×10^{-6}
C64–C128–C128–C128	9.01×10^{7}	4.40×10^{-5}	3.60×10^{6}	2.77×10^{-6}
C128–C128–C128–C128	1.47×10^{8}	2.70×10^{-5}	5.90×10^{6}	1.70×10^{-6}
C128–C256–C256–C256	3.51×10^{8}	1.10×10^{-5}	1.40×10^{7}	7.13×10^{-7}

Supplementary Table 4. Number of operations in the convolutional SPDNN models. The table displays the number of multiply–accumulate (MAC) operations and dot products (DPs) in the SPDNN models. $N_{\text{MAC}}^{\text{total}}$ ($N_{\text{DP}}^{\text{total}}$) represents the total number of MAC operations (dot products) in the entire SPDNN models, including all the convolutional layers and two linear layers. $N_{\text{MAC}}^{\text{out}}$ ($N_{\text{DP}}^{\text{out}}$) represents the number of MAC operations (dot products) in the output layer, which is the only layer implemented with a "high SNR". For the $400 \to 10$ output layer, $N_{\text{MAC}}^{\text{out}} = 4000$ and $N_{\text{DP}}^{\text{out}} = 10$. The portion of the high-SNR output layer's operations relative to the entire model's operations in terms of MAC operations (dot products) is presented in the third (fifth) column. "CN_{chan}" denotes an SPD convolutional layer with N_{chan} output channels.

C. Additional classification tasks

Apart from the benchmark MNIST and CIFAR-10 classification tasks, we added additional tests for KMNIST ("Kuzushiji MNIST") [26] and FashionMNIST [27] to further demonstrate the capabilities of our SPDNN models. To ensure fair and straightforward comparisons, we chose to test these tasks using the same structure we used for MNIST classification tasks, specifically the MLP with $784 \to 400 \to 10$. We compared the following four different models:

- A deterministic MLP with one hidden layer of 400 neurons, $784 \to 400 \to 10$. The weights are real-valued. The activation function is ReLU.
- A coherent MLP-SPDNN with one hidden layer of 400 neurons, $784 \to 400 \to 10$. The weights are real-valued. This model is the same as the one shown in Figure 4d in the main text ($N = 400$).
- An incoherent MLP-SPDNN with one hidden layer of 400 neurons, $784 \to 400 \to 10$. The weights are non-negative. This model is the same as the one shown in Figure 3b in the main text (MLP, 1 hidden layer).
- A linear classifier. The weights are real-valued.

Using a training process similar to that for the MNIST classification task (Supplementary Note 2 A), we optimized these SPDNN models with both incoherent and coherent optical setups. The blue dashed line represents the deterministic model with the same MLP architecture as the SPDNNs. The red dashed line represents the linear classifier. These lines set the upper and lower bounds of test accuracy, respectively, for each individual classification task.

By varying the number of shots per activation K in these SPDNN models, we observed trends consistent with those seen in the MNIST task (Supplementary Figure 6), although some small differences exist among these tasks. For

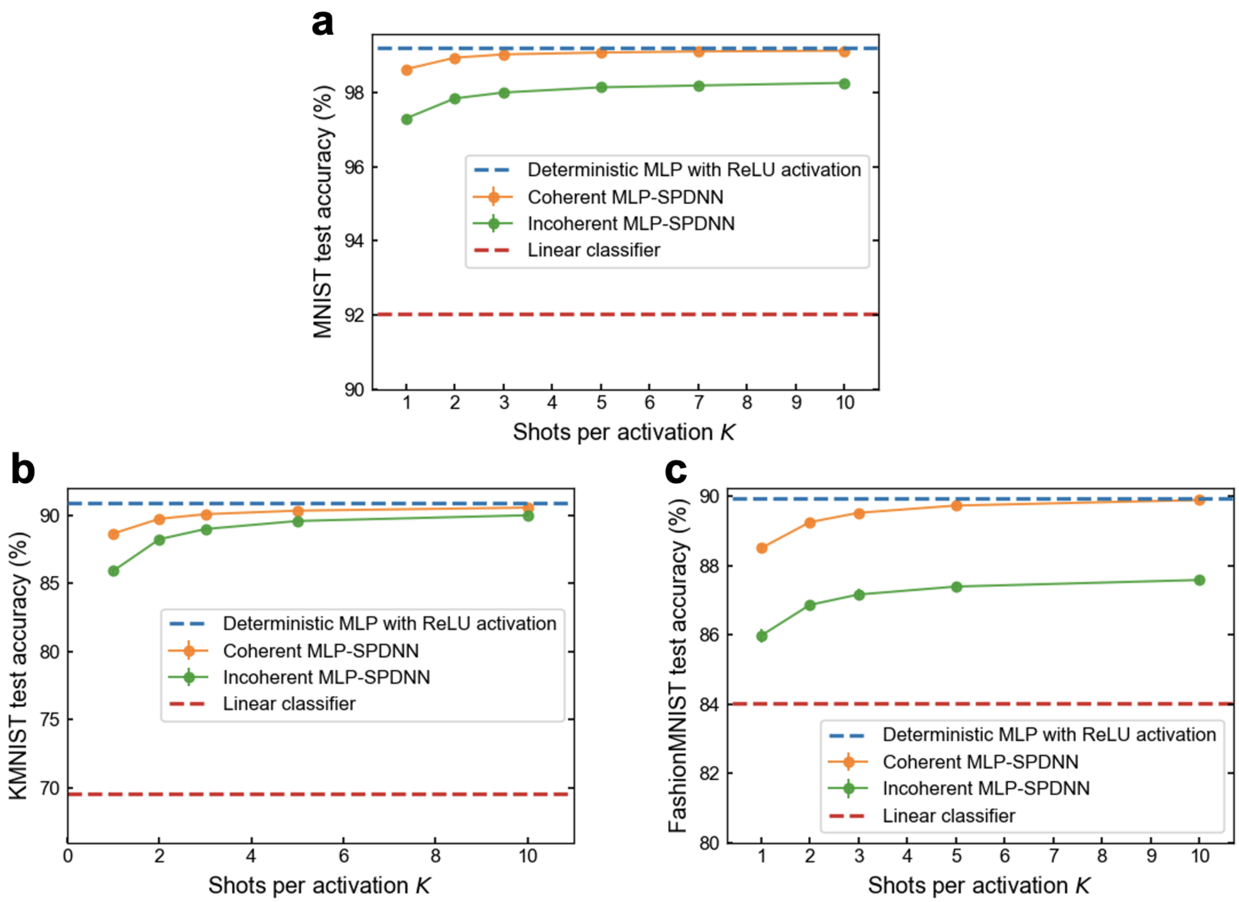

Supplementary Figure 6. Comparison of performance of different models on three image classification tasks. The deterministic model and both coherent and incoherent SPDNNs share the MLP architecture of $784 \to 400 \to 10$. The x-axes represent the number of shots per activation K in the hidden layer of the corresponding SPDNN model. These models are trained for **a,** MNIST, **b,** KMNIST, and **c,** FashionMNIST classification tasks, respectively.

example, the test accuracy for coherent and incoherent MLP-SPDNNs tends to converge for the KMNIST classification task with increasing shots per activation K. However, this convergence is less pronounced for the other two tasks.

These additional results illustrate that our SPDNN models are effective across various tasks. They demonstrate that our highly stochastic SPDNN models can achieve reliable performance comparable to deterministic models.

Part II

Experimental setup

Supplementary Note 3. INCOHERENT OPTICAL MATRIX-VECTOR MULTIPLIER

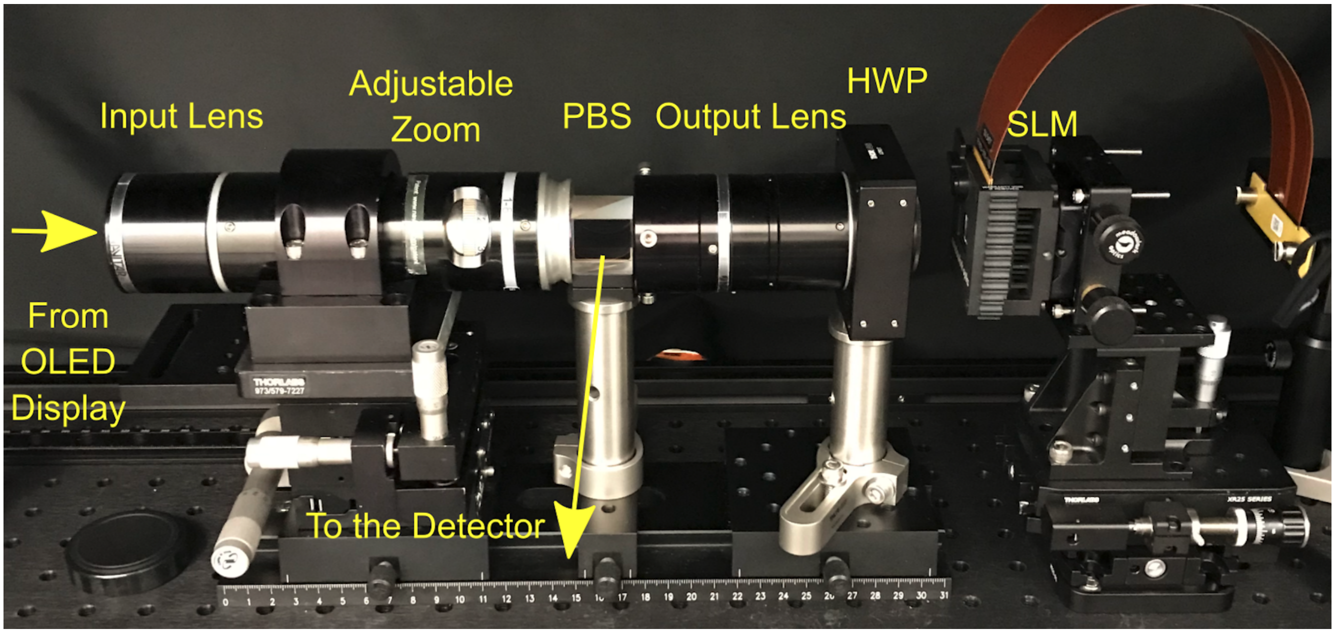

Supplementary Figure 7. A photo of the experimental setup. Input light source (OLED display) and detection parts are not included in this photo. PBS: polarizing beam splitter; HWP: half-wave plate; BPF: band-pass filter; SLM: spatial light modulator.

The optical matrix-vector multiplier (optical MVM) setup is based on the setup designed in [28]. It consists of an array of light sources, a zoom lens imaging system, an intensity modulator, and a photodetector. We used an organic light-emitting diode (OLED) display of a commercial smartphone (Google Pixel 2016 version) as the light source for encoding input vectors. The OLED display consists of a 1920×1080 pixel array, with individually controllable intensity for each pixel. There are pixels of three colors on the display, only the green pixels (light wavelength of ~ 532 nm) are used in the experiment. The green pixels are arranged in a square lattice with a pixel pitch of 57.5 µm. A reflective liquid-crystal spatial light modulator (SLM, P1920-500-1100-HDMI, Meadowlark Optics) was combined with a half-wave plate (HWP, WPH10ME-532, Thorlabs) and a polarizing beam splitter (PBS, CCM1-PBS251, Thorlabs) to perform intensity modulation as weight multiplication. The SLM has a pixel array of dimensions 1920×1152, with individually controllable transmission for each pixel. Each pixel has the size of 9.2×9.2 µm. A zoom lens system (Resolv4K, Navitar) was used to image the OLED display onto the SLM panel (Supplementary Figure 7). The intensity-modulated light field reflected from the SLM was further de-magnified and imaged onto the detector, by a telescope formed by the rear adapter of the zoom lens (1-81102, Navitar) and an objective lens (XLFLUOR4x/340, Olympus). An additional band-pass filter (BPF, FF01-525/15-25, Semrock) and polarizer (LPVISE100-A, Thorlabs)

were inserted into the telescope in order to reduce the bandwidth and purify the polarization of the light reflected by the PBS. A scientific CMOS camera (ORCA-Quest qCMOS Camera C15550-20UP) is used to measure the light intensity, as well as single-photon detection. The qCMOS camera has 4096×2304 effective pixels with the size of 4.6×4.6 µm.

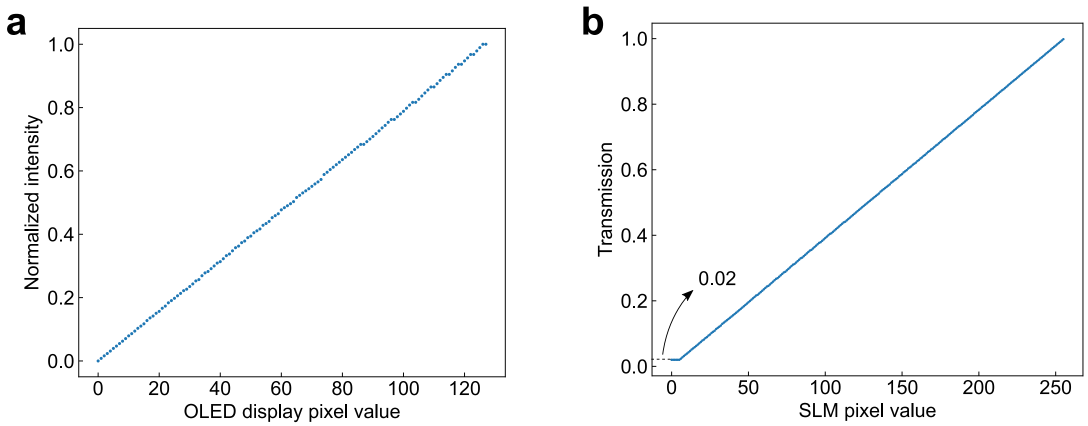

Supplementary Figure 8. Look-up tables (LUTs) of the components in the incoherent optical matrix-vector multiplier (optical MVM) setup. a, The 7-bit LUT of the OLED display to control the pixel intensity. **b,** The 8-bit LUT of the SLM for intensity modulation. The minimum transmission was measured to be $\sim 2\%$ of the maximum transmission.

During the computation of a vector-vector dot product, the value of each element in either vector is encoded in the intensity of the light emitted by a pixel on the OLED and the transmission of an SLM pixel. Via the imaging system, each pixel on the OLED display is aligned to a corresponding pixel on the SLM, where element-wise multiplication takes place by the intensity modulation. The modulated light intensity from pixels in the same vector is then focused on the detector to sum up the element-wise multiplication values to be the dot product result. Since the light is incoherent, only non-negative values can be represented. Matrix-vector multiplication is realized by doing a batch of this kind of vector-vector multiplications in parallel, either multiplexed in space or time.

OLED pixels feature a high extinction ratio and high dynamic range in intensity, which are ideal for characterizing the accuracy of vector-vector dot products. A commercial-grade integrated OLED panel with a high pixel count is readily available at a low cost, which made it possible to encode very large vectors that were essential for demonstrating vector-vector dot products on our setup. The true darkness of OLED pixels allowed us to achieve high dynamic range in intensity modulation and to reduce noise caused by background light pollution. The intensity of each individual pixel can be controlled independently with 256 (8-bit) control levels. However, since the actual output intensity was not linear with the pixel control level, we calibrated a linear look-up table (LUT) that contains 124 distinct intensity levels (~7 bits, Supplementary Figure 8a). We converted a phase-only SLM into an intensity modulator with a half-wave plate (HWP) and a polarizing beam splitter (PBS). The SLM pixels are made of birefringent liquid crystal layers, whose refractive index can be tuned by applying voltage across them. By controlling the refractive index of extraordinary light, the SLM pixels introduce a phase difference between the extraordinary and ordinary light, whose

polarizations are perpendicular to each other. When a PBS and HWP were placed in front of a reflective SLM, the light field passed the components twice, once during the trip towards the SLM and once after being reflected by the SLM (Supplementary Figure 6). One of the functions of PBS was to separate the output from the input light: the input light (incident to the SLM) was horizontally polarized and transmitted by the PBS, while the output light (reflected from the SLM) was vertically polarized, and therefore reflected by the PBS. The other function of the PBS is to convert the polarization state of the output light to its amplitude: the light modulated by the SLM was in general elliptically polarized, controlled by the phase difference. The amplitude of the light field (and intensity in this case too) was modulated by selecting only the vertical component of the SLM-modulated light at the output port of the PBS. The HWP was placed with its fast axis rotated 22.5 degrees from the extraordinary axis of the SLM such that the intensity transmission could be tuned from 0 to 100%. In the experiment, each of the SLM pixels can be independently controlled for intensity modulation with a 256 (8-bit) LUT (Supplementary Figure 8b). The maximum extinction ratio of the transmission intensity was measured to be ∼50. Alternatively, instead of using a phase-modulation SLM, the intensity modulator can be more compactly implemented with a monolithic LCD panel in a transmission geometry.

Supplementary Note 4. SINGLE-PHOTON DETECTION BY A SCIENTIFIC CMOS CAMERA

Single-photon detection is core to implementing an SPDNN. In our experiment, we use a scientific CMOS camera, the Hamamatsu ORCA-Quest qCMOS Camera, to realize the function of single-photon detectors [29]. CMOS cameras usually cannot detect single photons due to the relatively high readout noise compared to the signals induced by individual photons. The ORCA-Quest qCMOS camera, however, has well-controlled readout noise as low as 0.3 equivalent photoelectrons. This makes viewing the individual spikes of photon response possible on the output of the camera. An example of the distribution of pixel values from the camera is shown in Supplementary Figure 9a. These pixel values are from a sequence of frames collected with some intensity of input light. The output pixel values have a digital bias of ≈ 200, and the analog gain is ~ 7.94 pixel values per photoelectron. We can see the individual spikes corresponding to different numbers of detected photons, with the first peak referring to no detected photons. Due to readout noise of the camera, we can still see a near-Gaussian distribution around the peak value of each detected photon number. To do single-photon detection, a threshold can be set to determine if there is a photon (or more photons) detected. If the pixel value is larger than the threshold, we record a click; otherwise, there is no click. In this way, although the camera has already completed analog-to-digital conversion (ADC) before thresholding, the qCMOS camera can still emulate the function of a single-photon detector.

This camera has an overall quantum efficiency of $\sim 86\%$ at our working wavelength of 532 nm and a dark count rate of 0.006 photoelectrons per second per pixel at the working temperature of -35°C with air cooling. Due to the readout noise, the thresholding process may add additional errors because of the overlap of the signal peaks. Supplementary Figure 9b shows the pixel value distribution of dark frames without input signals. We can see that using the same threshold, there is a small tail of the distribution on the right side of the threshold. This small portion of the pixel values from dark frames would trigger a photon click as well, which further adds to the dark count rate. Similarly, the

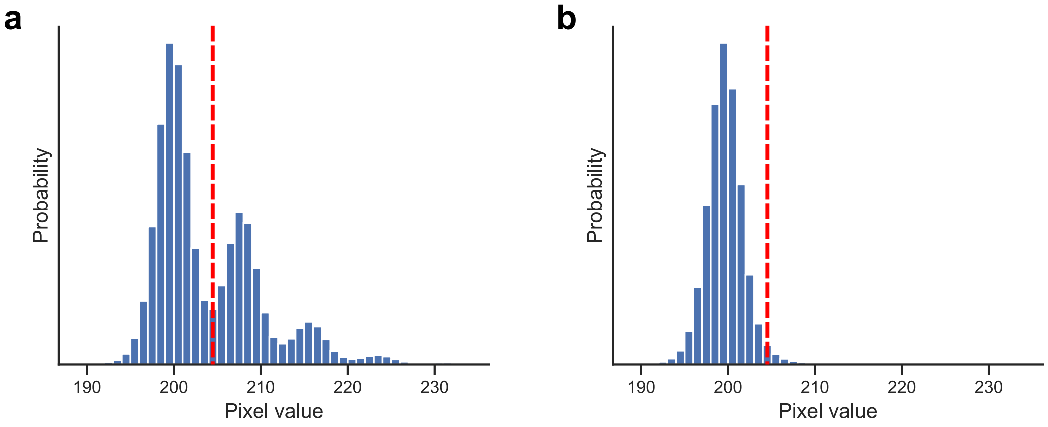

Supplementary Figure 9. Pixel value distributions of the ORCA-Quest qCMOS camera. a, An example of pixel value distribution in collected frames with input signal light of a few photons. The red dashed line indicates the threshold to tell if there is a photon click. We can see clearly that the discrete numbers of photons can be resolved in the individual peaks. **b,** Pixel value distribution of the dark frames without input light.

output pixel values from detected photons also have a small probability to fall in the "no click" region, which makes the effective photon detection efficiency a bit lower. In our experiment, we calibrated the qCMOS camera in the single-photon detection mode and found that the effective dark count rate of around 0.01 photoelectrons per second per pixel, and the effective photon detection efficiency to be 68%, on average. Note that there are also variations among different pixels. In the experiment implementation, only one single pixel is used for each vector-vector dot product. We will see that the photon detection efficiency and dark counts do not significantly influence the results, as discussed in Supplementary Note 10.

Supplementary Note 5. VALIDATION OF THE OPTICAL VECTOR-VECTOR MULTIPLICATIONS

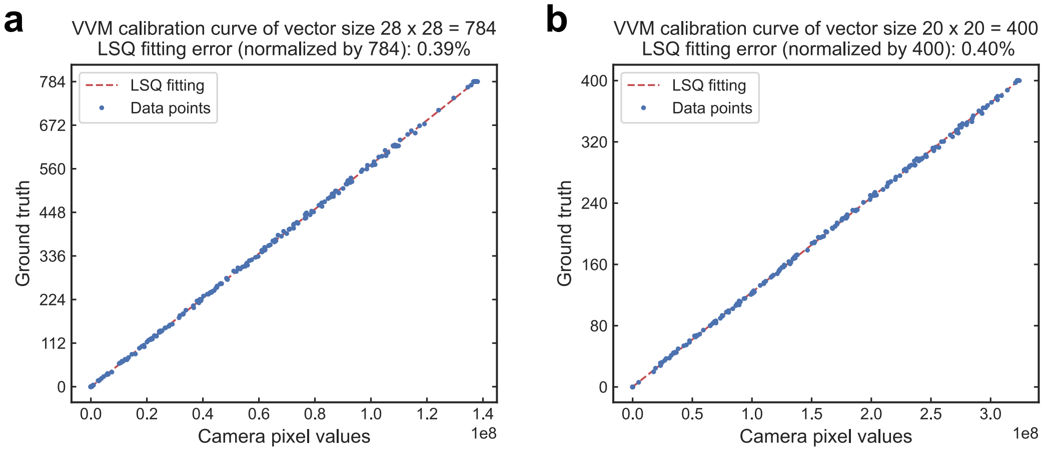

Supplementary Figure 10. Calibration curves of vector-vector multiplication (VVM) precision in the setup. Panel **a** (**b**) shows the VVM calibration of the vector size of 784 (400), which were used for the hidden (output) layer in the optical implementation of the SPDNN model with a structure of 784 $\to$ 400 $\to$ 10. The calibration curves (red dashed line) were obtained using the least-squares regression method and the data points are collected with a long exposure time to eliminate the photon noise.

The major part of computation in the ONN implementation is the linear operations. The accuracy of matrix-vector multiplication is essential in a successful ONN inference. In this section, we calibrate the accuracy of our optical MVM. We use the setup with either single-photon detection or conventional intensity measurement that involves a much higher intensity. Focusing the lights to one pixel of $\sim$ 5 µm is challenging, which reduces the dot product precision slightly. However, as we will see in Supplementary Note 10, the SPDNN models are very robust to this amount of errors.

To generate a test dataset representative of general dot products, we randomly generated vector pairs $\vec{x}$ and $\vec{w}$ based on natural scene images from the STL10 dataset. Each vector was generated from a single color channel of one or more images patched together, depending on the target vector size (each image of size $L \times L$ contributes $N = L^2$ elements to the vector). We chose natural images since they are more representative of the inputs in image classification with globally inhomogeneous and locally smooth features. To adjust the sparsity of the vectors, different thresholds were applied to the image pixel values such that the dot product results cover a wider range of possible values. This was achieved by shifting the original pixel values (float point numbers normalized to the range 0-1) in the entire image up or down by a certain amount, unless the value was already saturated at 1 (maximum) or 0 (dark). For example, a shift of -1 would make the whole image dark. A shift of +0.2 would make all the pixel values that were originally larger than 0.8 saturated, and would increase all other pixel values by 0.2. This method allowed us to tune the overall intensity of the modulated images without losing the randomness of the distribution.

Calibration curves of vector-vector dot product results are shown in Supplementary Figure 10. The results are averaged over a large number of repetitions to get rid of the photon noise to see the systematic errors in the optical MVM. The vectors are randomly generated to cover the full range of the light intensity from the minimum to maximum transmission, as discussed in [28]. The vector size is 28×28, which is equivalent to the size of the first layer in MNIST classification.

Supplementary Note 6. VALIDATION OF THE SPD ACTIVATION FUNCTION

To validate the SPD activation function in the SPDNN implementation, we need to consider not only the precision of the linear operations, but also the non-linear activation function. As the incident light onto the qCMOS camera is attenuated to just a few photons, the photon noise becomes significant and the measurement less accurate. To address this, we first measure a higher light intensity with long exposure times and estimate the exact light intensity with a shorter exposure time using the ratio of exposure times. We then use the shorter exposure time to perform single-photon detection to output a photon click (value 1) or no photon click (value 0). The probability of a photon click is estimated by averaging over a large number of repetitions. The intensity is tuned by adjusting both the exposure time and neutral density (ND) filters that attenuated the light. The expected theoretical curve is also plotted for comparison. The results are shown in Supplementary Figure 11.

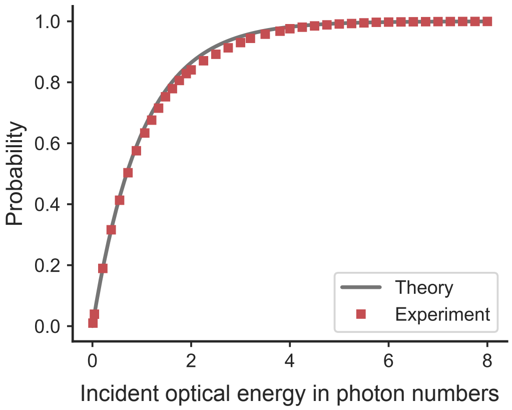

Supplementary Figure 11. Validation of the SPD activation function. The theory curve is the expected function of $f(\lambda) = 1 - e^{-\lambda}$ with the intensity λ in photon numbers. The experiment data was taken by the Hamamatsu ORCA-Quest qCMOS camera.

Part III

Implementation of SPDNNs

Supplementary Note 7. ADAPTATION TO EXPERIMENTAL LIMITATIONS

The implementation of SPDNNs on an optical MVM can be challenged by experimental restrictions that affect the precision of the network inference. Some of these limitations include the non-negative encoding resulting from the use of an incoherent light source and limitations in the precision of the setup. In this section, we describe how these limitations can be addressed to successfully implement SPDNNs on our setup. As discussed in Supplementary Note 3, our incoherent optical MVM has systematic errors in the dot-product results, even in the absence of photon noise. Additionally, the SLM used in the system has a finite extinction ratio of approximately 50 (Supplementary Figure 8b). These limitations present a significant challenge in the implementation of the SPDNNs because, in the models, both the input vectors and weights have many small values close to 0. This is problematic because, within the full range of 0 to 1, having a minimum value of 0.02 instead of 0 has a non-trivial effect on the accuracy of the dot product calculation. These small values are accumulated over many elements, leading to a relatively large value, compared to the final dot product result. As a result, the performance of the SPDNNs is severely impacted by these limitations.

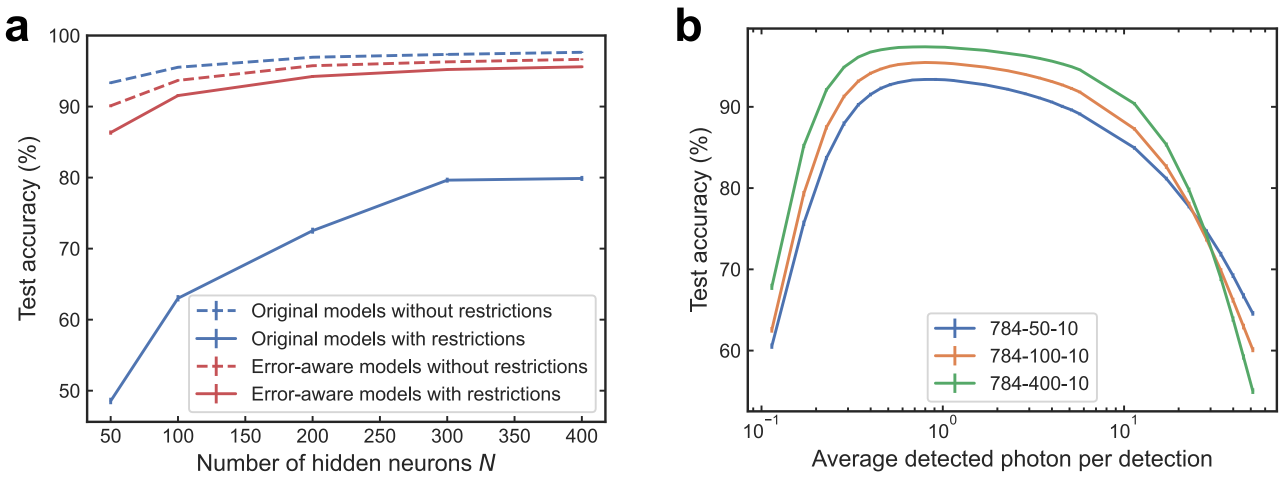

Supplementary Figure 12. Simulation of SPDNN performance with different experimental settings. **a,** MNIST test accuracy of SPDNN models under experimental restrictions. The models have a structure of $784 \rightarrow 400 \rightarrow 10$ ($N = 400$, $K = 1$). **b,** MNIST test accuracy as a function of the input light intensity. The intensity was varied by adjusting the range of input values using a constant factor. Both panels show results obtained with an incoherent setup and a single shot of SPD readout per activation ($K = 1$).

Supplementary Figure 12a demonstrates the results of implementing the neural network models using the real LUTs from our setup. The test accuracy significantly drops, making the experimental implementation a failure. To address this issue, we used error-aware training techniques (as discussed in [30]) to train our models with an understanding of these experimental restrictions. During the error-aware training process, the real LUTs were used in the implemen-

tation of the models. The results of this error-aware training are shown in the red curves in Supplementary Figure 12a. It can be seen that, with error-aware training, the SPDNN models are highly robust to changes in input range, especially with a relatively large number of hidden neurons.

Conventional ONN inferences can operate effectively at various light intensity levels, as long as the intensity is sufficiently high to suppress photon noise and maximize detection precision. These systems can, in principle, integrate arbitrarily high light intensities to enhance detection precision. However, in the optical implementation of SPDNN inferences, the SPD activation function relies on the precise number of photons detected. As a result, controlling the operating intensity in the setup becomes crucial to ensure accurate quantization of the detected optical energy. Calibrating the intensity to the appropriate level for SPD activation function presents a challenge, especially considering the inherent significant noise in intensity measurements at low photon counts.

Despite these challenges, our simulation results demonstrate the robust performance of SPDNNs even with slight variations in the input intensities. We systematically varied the input intensity across a range from 0.1 to 100 times the original expected intensity that was used during training. Supplementary Figure 12b illustrates that the model's performance remains stable within a wide range of intensities. The test accuracy remains nearly consistent, even when the input energy deviates significantly from the original training intensity. This observed stability highlights the resilience of SPDNNs to variations in input intensity levels. It further suggests that these SPDNN models can be successfully implemented with lower photon budgets, which is promising for practical applications where minimizing optical energy usage is desirable.

Supplementary Note 8. OPTICAL IMPLEMENTATION OF SPD ACTIVATIONS

In this section, we demonstrate the experimental implementation of SPD activation functions in SPDNN inferences. The weight matrix of the first layer $W^{(1)}$ in the SPDNNs was displayed on the OLED screen, with each element encoded as the intensity of a corresponding pixel. The models used in experiments are trained to account for the limitations of the experimental setup, as discussed in Supplementary Note 7. In Supplementary Figure 13, the weights of the model with $N = 100$ hidden neurons are depicted. The two-dimensional arrangement of each weight vector block mirrors the visual representation on the OLED display, which matches each pixel of the MNIST input images. The MNIST test images were displayed on the SLM, where the pixels were aligned with those on the OLED display. The transmission of each SLM pixel is determined by the values of the corresponding pixels in the input image. After passing through the SLM, the modulated intensity of each pixel is then combined through the imaging system to perform an optical fan-in process, and the dot product result is obtained by detecting the accumulated optical energy.

As described in Supplementary Note 4, the qCMOS camera serves as a single-photon detector (SPD). The modulated light is recorded by the qCMOS camera, and the SPD activation function is applied during this single-photon detection process. A single pixel on the qCMOS camera is used and emulates a single-photon detector by applying a threshold on the output pixel values. As shown in Supplementary Figure 9, the output signal is primarily discrete in individual photon numbers, with only slight mixing due to readout noise from the electronic apparatus. Robustness to the false clicks due to the mixing will be discussed in Supplementary Note 10. To control the input light intensity, either the

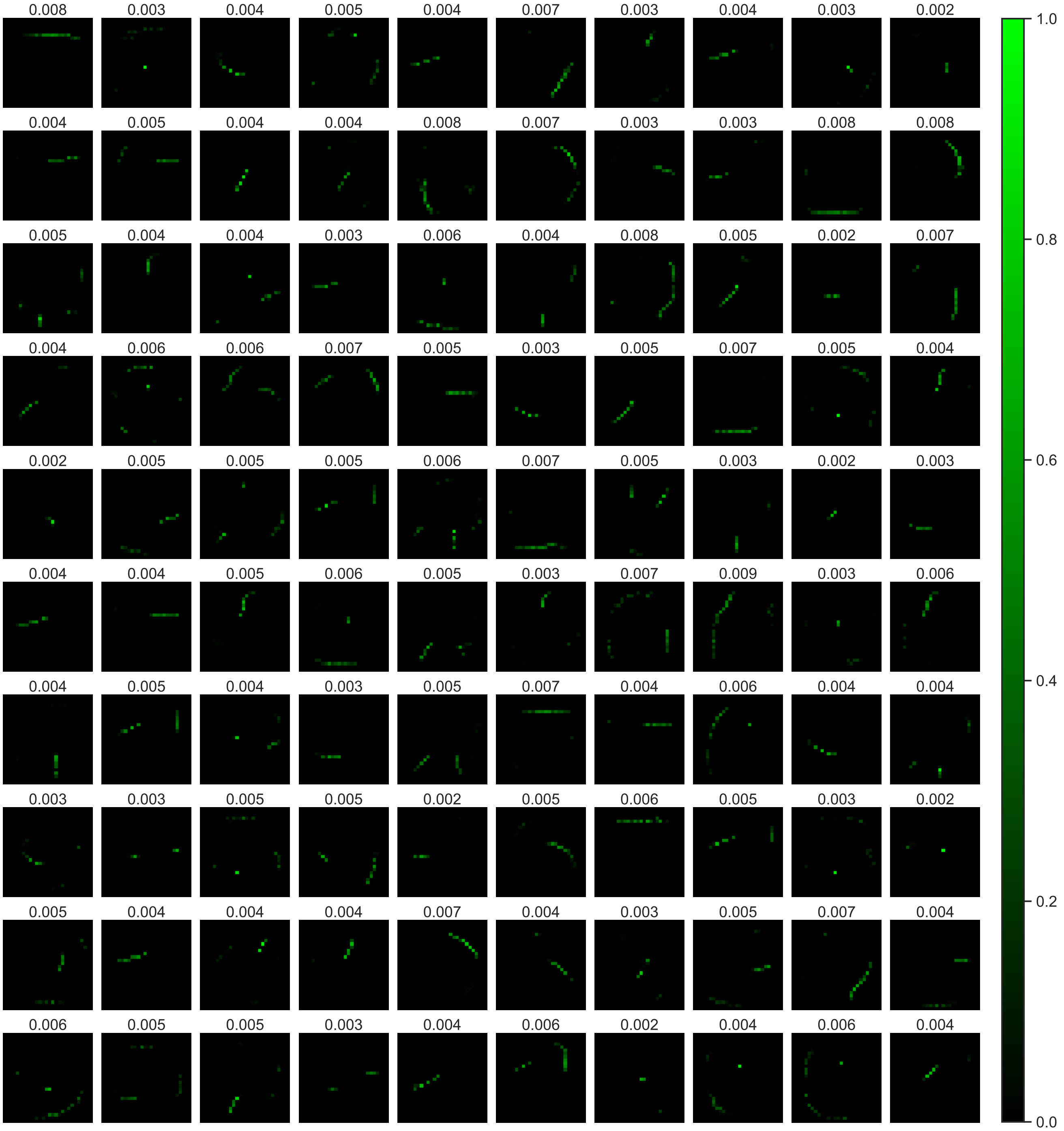

Supplementary Figure 13. An example of weight values to be displayed on the OLED display during the experiment. This model has $N = 100$ hidden neurons, and we display the weight matrix $W^{(1)}$ of the first layer (with dimensions 100×784). Each block represents a row vector in $W^{(1)}$ containing 784 elements. These column vectors are rearranged to form a 2D block with dimensions 28×28, matching the original shape of the MNIST input images. The 100 rows in $W^{(1)}$, corresponding to the 100 hidden neurons in the neural network, are arranged in a 10×10 grid to be visualized. The weight values have been normalized to a range of 0 to 1 to fit the intensity range of the OLED display. The average value of each block is indicated at the top. The color map used in the plot has been selected to emulate the actual color on the OLED display, as only green pixels are utilized (~ 532 nm), thereby presenting what could be observed on the OLED display in the experimental setup.

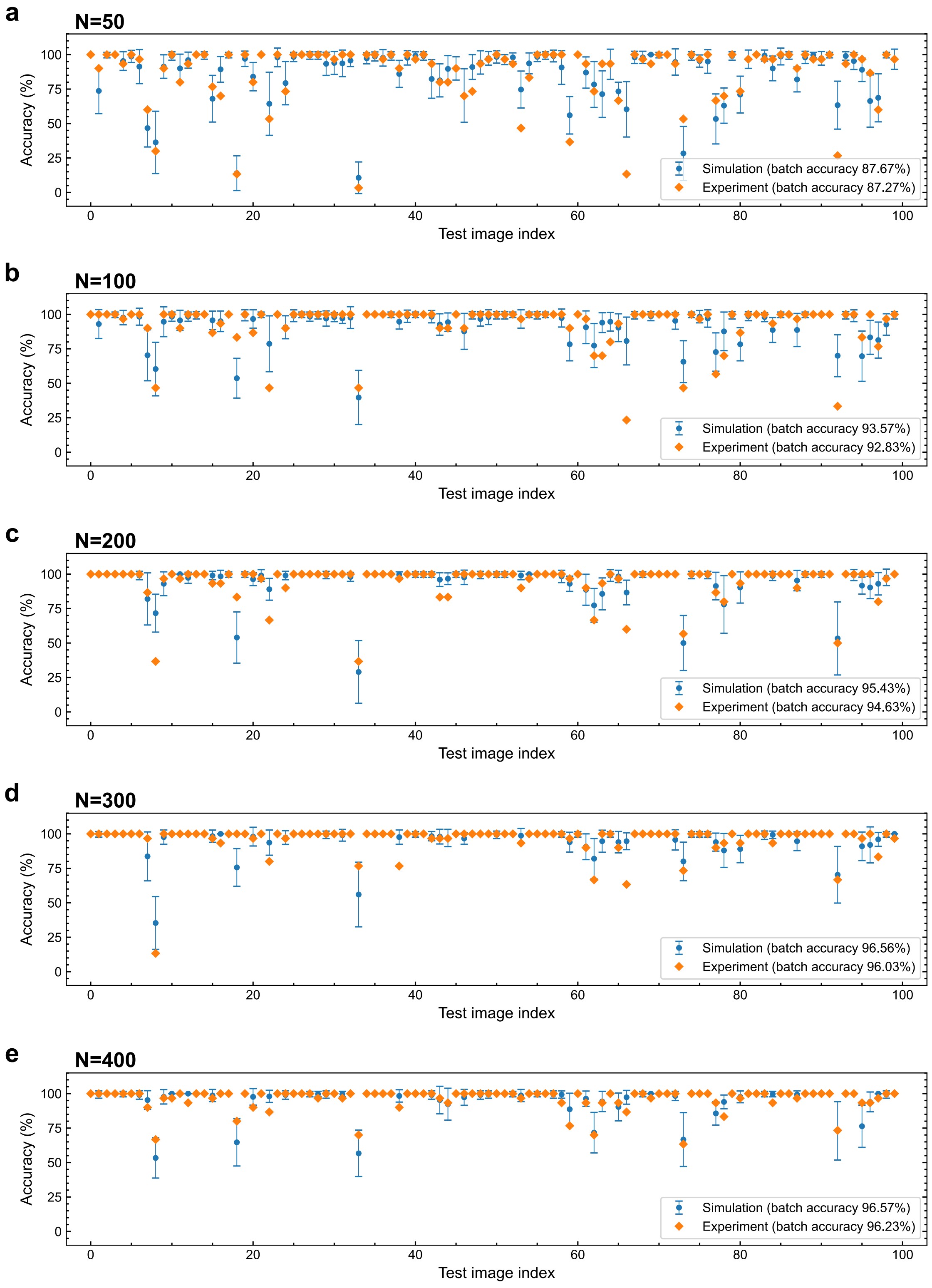

Supplementary Figure 14. Test accuracy of individual images with 1 SPD measurement per activation ($K = 1$). The MLP-SPDNN models with a varying number of hidden neurons N from 50 to 400 (panels **a**–**e**) were evaluated using a single shot per SPD activation ($K = 1$). Test accuracy was evaluated for each individual image, and each data point represents the average accuracy obtained from 30 inferences. We have not plotted error bars for experimental data, but the variance is directly related to the data shown: since the outcome of each inference is a Bernoulli random variable with p, the variance is $p(1-p)$. The accuracy values in the legends are averaged over all test images. The simulation results used the same SPDNN models as in the experiment and considered the experimental restrictions. The error bars were calculated by repetitions of the whole process (average accuracy of 30 inferences).

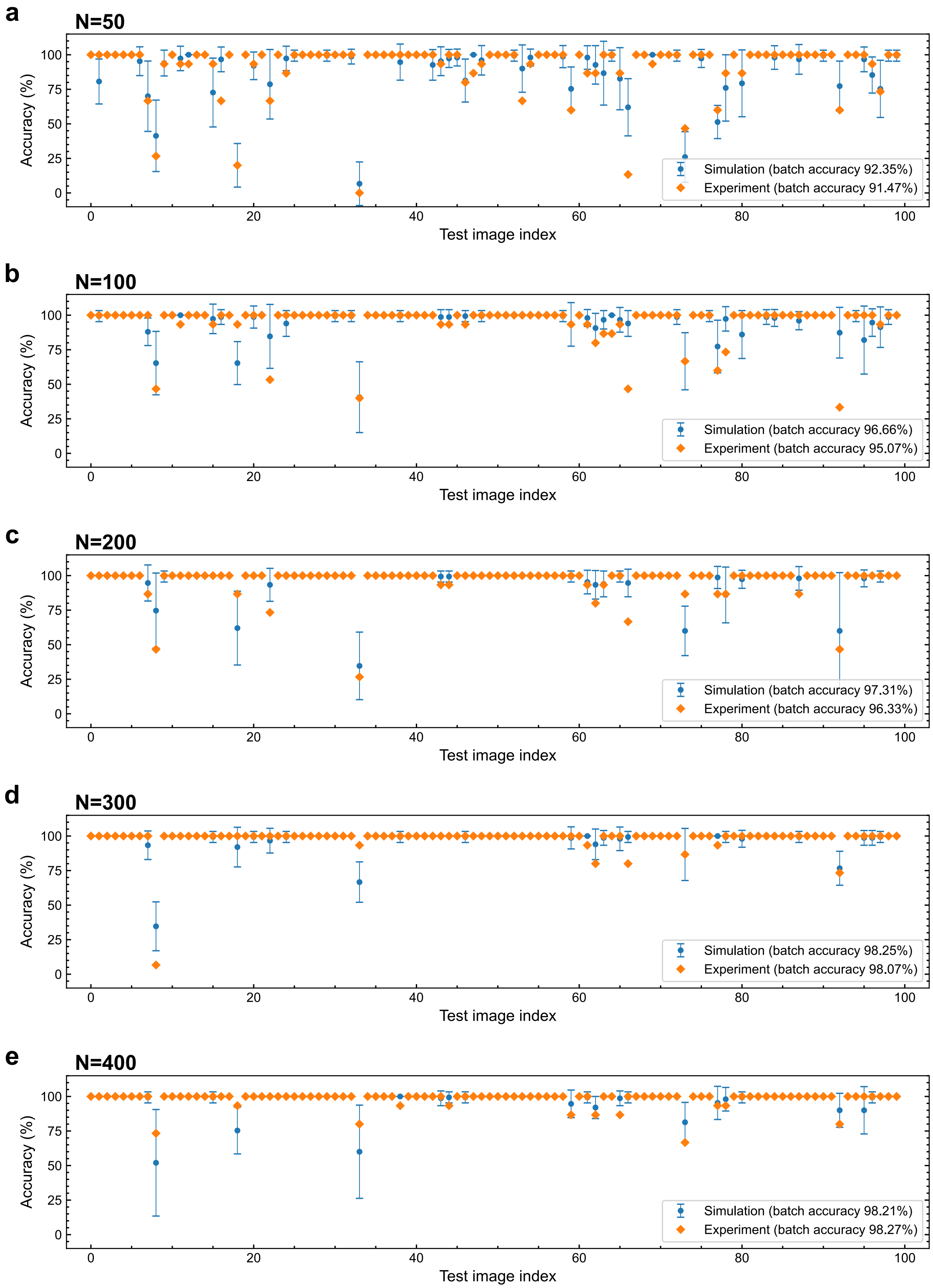

Supplementary Figure 15. Test accuracy of individual images with 2 SPD measurements per activation ($K = 2$). The MLP-SPDNN models with a varying number of hidden neurons N from 50 to 400 (panels **a**–**e**) were evaluated using 2 shots per SPD activation ($K = 2$). Test accuracy was evaluated for each individual image, and each data point represents the average accuracy obtained from 15 inferences. We have not plotted error bars for experimental data, but the variance is directly related to the data shown: since the outcome of each inference is a Bernoulli random variable with p, the variance is $p(1-p)$. The accuracy values in the legends are averaged over all test images. The simulation results used the same SPDNN models as in the experiment and considered the experimental restrictions. The error bars were calculated by repetitions of the whole process (average accuracy of 15 inferences).

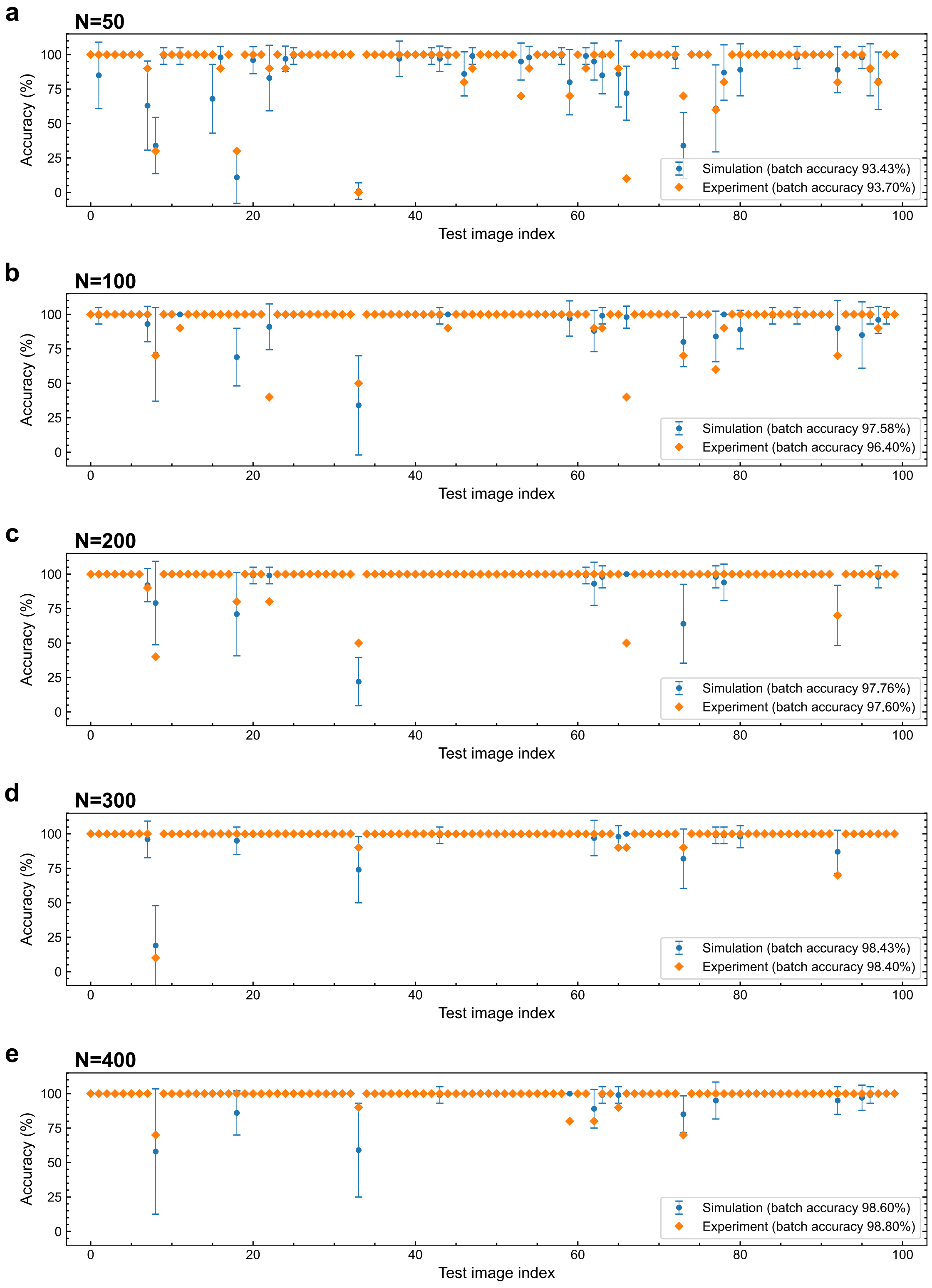

Supplementary Figure 16. Test accuracy of individual images with 3 SPD measurements per activation ($K = 3$). The MLP-SPDNN models with a varying number of hidden neurons N from 50 to 400 (panels **a**–**e**) were evaluated using 3 shots per SPD activation ($K = 3$). Test accuracy was evaluated for each individual image, and each data point represents the average accuracy obtained from 10 inferences. We have not plotted error bars for experimental data, but the variance is directly related to the data shown: since the outcome of each inference is a Bernoulli random variable with p, the variance is $p(1 - p)$. The accuracy values in the legends are averaged over all test images. The simulation results used the same SPDNN models as in the experiment and considered the experimental restrictions. The error bars were calculated by repetitions of the whole process (average accuracy of 10 inferences).

exposure time is varied or light attenuation can be achieved through the use of neutral density filters, or a combination of both, depending on realistic experimental settings. A typical exposure time value is ~ 1 ms for one shot of SPD measurement.

Due to the inherent stochastic nature of SPDNN models, the output from different repetitions of the same test image and weight matrix varies. To assess the performance of the model, it is necessary to repeat the inference many times to represent the distribution of the output. This will give us a better understanding of the model's behavior and its accuracy. In the experimental setup, multiple repetitions of the inference were obtained by capturing multiple frames with the camera. Each repetition is identified by a unique frame index number. Note that this repetition includes the entire inference process. Each repetition of inference for a given input image involves $N \times K$ SPD measurements, with K shots of SPD measurements for each of the N neuron activations in the hidden layer.

In our experiments, we collected a set of 30 frames for each test image and weight matrix combination. Each frame provides us with a measurement of the stochastic binary output values produced by the SPD activation function, effectively serving as one-shot activations for each hidden neuron in the model. The activations for different frames, distinguished by different frame indices, are considered as distinct repetitions of the inference process.

As explained in Supplementary Note 1 A, we can improve the precision of the inference by employing multiple shots per activation. With K shots, we average the K binary values to obtain the actual activation value during that inference. In our experiment, every K frames are averaged to obtain the activation value during a K-shot inference. As each frame collected during the experiment is independent and identically distributed, the specific sequence or arrangement of the frame indices has no impact on the resulting outcome. Subsequently, the activation values were used as inputs to the output linear layer, performed digitally with full precision, to calculate the final output and make predictions for the label of the test image. The test accuracy results can be found in Supplementary Table 5 and Figure 3 in the main text.

Our experimental results show that the collected SPD activations are capable of producing similar test accuracy results to those obtained from simulation. However, simply having similar accuracy values may not be a sufficient indicator of a faithful implementation. To validate this further, we compare the prediction accuracy of individual test images between the experimental and simulated results. The results obtained from 1-shot inferences $K = 1$ are presented in Supplementary Figure 14, where we use each frame as a separate repetition of the inference process, with a total of 30 repetitions performed. For each repetition, if the prediction made was accurate, it was recorded as a 1; otherwise, it was recorded as a 0. To visualize the distribution of the output accuracy, we then calculated and plotted the mean values and standard deviations of the test accuracy based on the 30 repetitions.

We conducted simulations of the same inference process on a digital computer using the same models and input images. To ensure a closer simulation to reality, we also incorporated realistic experimental restrictions, such as the limited extinguish value of the SLM, the dynamic range and precision of the LUT in both the SLM and the OLED display, and the systematic errors in the optical MVM.

Similarly, we examined the results of the inferences with $K = 2$ ($K = 3$) shots per activation, which are illustrated in Supplementary Figure 15 (16). In this setup, we combine every 2 (3) frames to be averaged to compute a neuron activation, and we repeated this process 15 (10) times to obtain the final results.

By comparing the simulation results with the experimental results obtained from the collected SPD activation values, we aimed to validate the performance of the latter. The comparison revealed that, for the majority of input

images, the predictions are highly resilient to the inherent stochasticity in the model. Interestingly, the results are not as unpredictable as one might expect, as a closer examination shows that most of the errors stem from a limited number of specific input images (see Supplementary Figure 14–16).

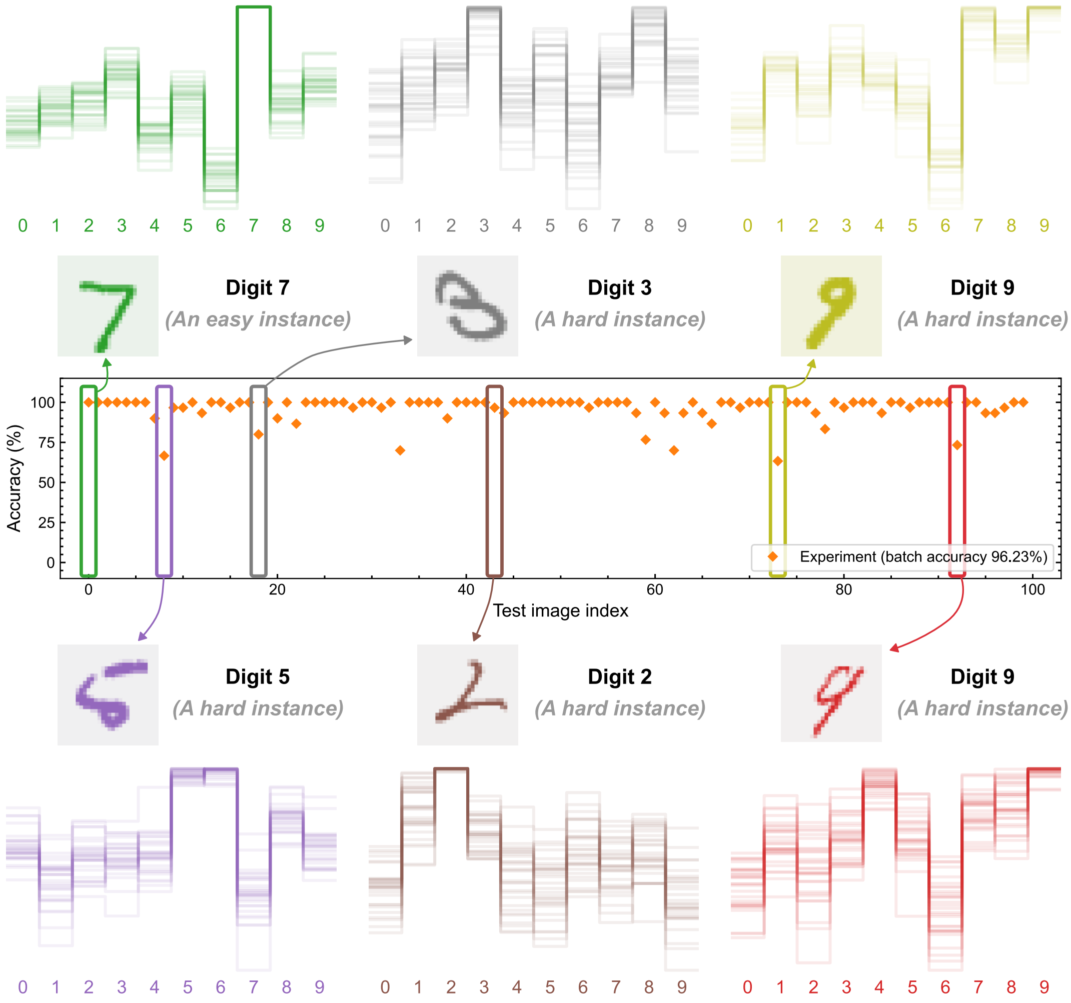

Supplementary Figure 17. Visualization of the output vectors of the SPDNN with given input images. In this figure, the SPDNN model has $N = 400$ hidden neurons and $K = 1$ shot of SPD measurement per activation (Supplementary Figure 14e). The prediction of a single inference of a particular test image is stochastic and is either correct or incorrect. For each test image, we performed 30 inferences for each test image and report the average accuracy. We have not plotted error bars, but the variance is directly related to the data shown: since the outcome of each inference is a Bernoulli random variable with p, the variance is $p(1-p)$. The output vectors from the 30 repetitions of inference with the fixed corresponding image are plotted together, with a 10% transparency on the curves to show the density. These output vectors are computed from the experimentally collected SPD activations by performing the output layer digitally on a computer (Supplementary Table 5).

The close correspondence between the experimental and simulated results for these specific "problematic" input images further validates the reliability of our experimental implementation. Although the experimental results are slightly inferior to the simulation results, the distribution of accuracy per input image is highly comparable. In particular, input images that exhibit high sensitivity to the model's stochasticity tend to result in larger deviations in the experimental results, while input images that are robust to the model's stochasticity exhibit high accuracy

Model	$K = 1$	$K = 2$	$K = 3$	$K = 5$	$K = 10$
$784 - 50 - 10$	$87.3 \pm 2.5\%$	$91.5 \pm 2.2\%$	$93.7 \pm 1.8\%$	$95.0 \pm 0.0\%$	$95.0 \pm 0.8\%$
$784 - 100 - 10$	$92.8 \pm 1.9\%$	$95.1 \pm 1.5\%$	$96.4 \pm 1.2\%$	$96.7 \pm 0.7\%$	$97.7 \pm 0.5\%$
$784 - 200 - 10$	$94.6 \pm 2.0\%$	$96.3 \pm 1.5\%$	$97.6 \pm 1.3\%$	$98.3 \pm 0.9\%$	$98.3 \pm 0.9\%$
$784 - 300 - 10$	$96.0 \pm 1.5\%$	$98.1 \pm 0.9\%$	$98.4 \pm 1.1\%$	$98.7 \pm 0.5\%$	$98.7 \pm 0.5\%$
$784 - 400 - 10$	$96.2 \pm 1.5\%$	$98.3 \pm 1.3\%$	$98.8 \pm 0.9\%$	$99.2 \pm 0.9\%$	$99.7 \pm 0.5\%$

Supplementary Table 5. Test accuracy of the experimental SPD activations with different shots per activation K. The results are based on 30 repetitions of one-shot binary SPD activations collected for each model structure. The test accuracy was calculated by averaging K shots per activation, and the last layer was performed with full precision. The table displays the mean and standard deviation of the test accuracy obtained from $30/K$ repetitions of inference.

both in simulations and in experiments. These results provide strong evidence of the reliability of the experimental implementation and demonstrate the robustness and noise resilience of SPDNN implementations.

To further understand the characteristics of the stochastic neural-network inference, we examined the output vectors of each input test image. As depicted in Supplementary Figure 17, the 30 output vectors from different repetitions of each input image are plotted together to demonstrate the stochasticity in the neural network. These output vectors were computed by the experimentally measured SPD activations and digitally implemented output layer, with $N = 400$ hidden neurons and $K = 1$ shot of SPD measurement per activation (Supplementary Figure 14e). No additional operations were performed after the linear operation of the output layer (see Algorithm 2).

Each of the 10 values in the output vector corresponds to the classes in MNIST digit classification, ranging from 0 to 9, as indicated at the bottom. The curves of the 30 output vectors were plotted with 10% transparency to show the distribution density.

As shown in Supplementary Figure 14–16, most of the test images have very high accuracy and are predicted correctly by SPDNN with high certainty, such as image 0 of digit “7” (depicted in the upper left in Supplementary Figure 17). Despite the stochastic distribution of the output values among the 30 repetitions, the value of class “7” remains consistently higher than the other elements, resulting in a 100% test accuracy for this image (see Figure 1a in the main text).

We also examined these “problematic” images, such as image 8 of digit “5” (lower left), which is predicted to be digit “6” nearly half of the chance. This misclassification is not surprising to human observers, as the image shares features with both digits “5” and “6”. Interestingly, the output values for class 8 in this case are relatively high but not the highest, which also aligns with human intuition.

Similar phenomena can be found for the other “problematic” images as well, indicating that the model has indeed learned meaningful features from the dataset. These findings solidify the fact that stochastic neural networks can perform reliable deterministic classification tasks, and the inherent stochasticity in the model does not compromise its ability to make accurate predictions.

Supplementary Note 9. FULL-OPTICAL IMPLEMENTATION OF THE ENTIRE SPDNN MODELS

In this section, we showcase a full-optical implementation of a neural network by demonstrating the implementation of the last linear layer optically as well, using the SPD activation values obtained from the inference of the first layer. This provides a comprehensive illustration of the feasibility of optical implementation for the entire network. It is important to note that, in conventional binarized neural networks, the last layer is usually implemented using full precision, as demonstrated in previous studies such as [31–34]. Our results demonstrate that SPDNNs can be implemented entirely using optics with remarkably low energy requirements. This capability holds promise for further advancements, especially with the integration of coherent optical computing platforms, which will be discussed later.

Similar to the first layer, we use the same setup to perform the optical matrix-vector multiplication. The difference is that now we do not need to perform single-photon detection that has to control the light intensity at a few photons per detection. In fact, the inference of the last linear layer can be implemented just as the conventional ONNs, where we accumulate a sufficiently high number of photons to reach a high SNR for each detection. The collected SPD activation values, as described in Supplementary Note 8, are used as inputs to the last linear layer. In the experimental implementation, we choose the data from the model with $N = 400$ hidden neurons and $K = 5$ shots per activation. For the 30 frames of one-shot binary SPD activations, every 5 frames of them are averaged to obtain the 6 independent repetitions of the inference. The input activation values to be displayed on the SLM are shown in Supplementary Figure 18. The possible values for the 5-shot activations are 0, 0.2, 0.4, 0.6, 0.8, and 1. If the linear operation was performed in full precision on a computer, the mean test accuracy would be approximately 99.1%. To perform the linear operation with real-valued weight elements on our incoherent setup, we divide the weight elements into positive and negative parts. We perform the operation separately for each part and finally obtain the output value by subtracting the results with negative weights from those with positive weights. The two sets of weights to be projected onto the OLED display are shown in Supplementary Figure 19, where the ten blocks of weights correspond to the ten output nodes. This approach at least doubles the photon budget required for the last layer and has the potential to be optimized for greater energy efficiency. However, even with these non-optimized settings, our results demonstrate that the optical energy budget is already several orders of magnitude lower than the state-of-the-art ONN implementations.

In the implementation, we adjust the exposure time of the camera to control the optical energy per detection. In order to perform the inference on the 100 input images and 10 output nodes, along with 6 repetitions of the activation values and 2 sets of weights, we need to perform a total of $100 \times 6 \times 10 \times 2 = 12000$ vector-vector dot products, each with a size of 400. Each vector-vector dot product detection is repeated 100 times. The results are presented in Supplementary Table 6. The photons per detection of either positive or negative output are each averaged over $100 \times 6 \times 10 = 6000$ dot products. The total photons detected in the last layer per inference are averaged over the 100 input images and 6 repetitions, totaling $100 \times 6 = 600$ inferences. The standard deviation of the photon numbers are calculated based on the 100 repeated detections for each dot product. The total detected photons in a full inference is the sum of those in the last layer and the first layer. The average value of the binary activations collected for the $N = 400$ model is $\sim$0.52186, resulting in a total of $0.52186 \times 400 \times 5 \approx 1043.7$ detected photons per inference in the first layer, with 5 shots per activation. This number is then combined with the total detected photons in the last layer to obtain the overall photon count for a full inference. We can see that the photon budget can be reduced by 5 folds if

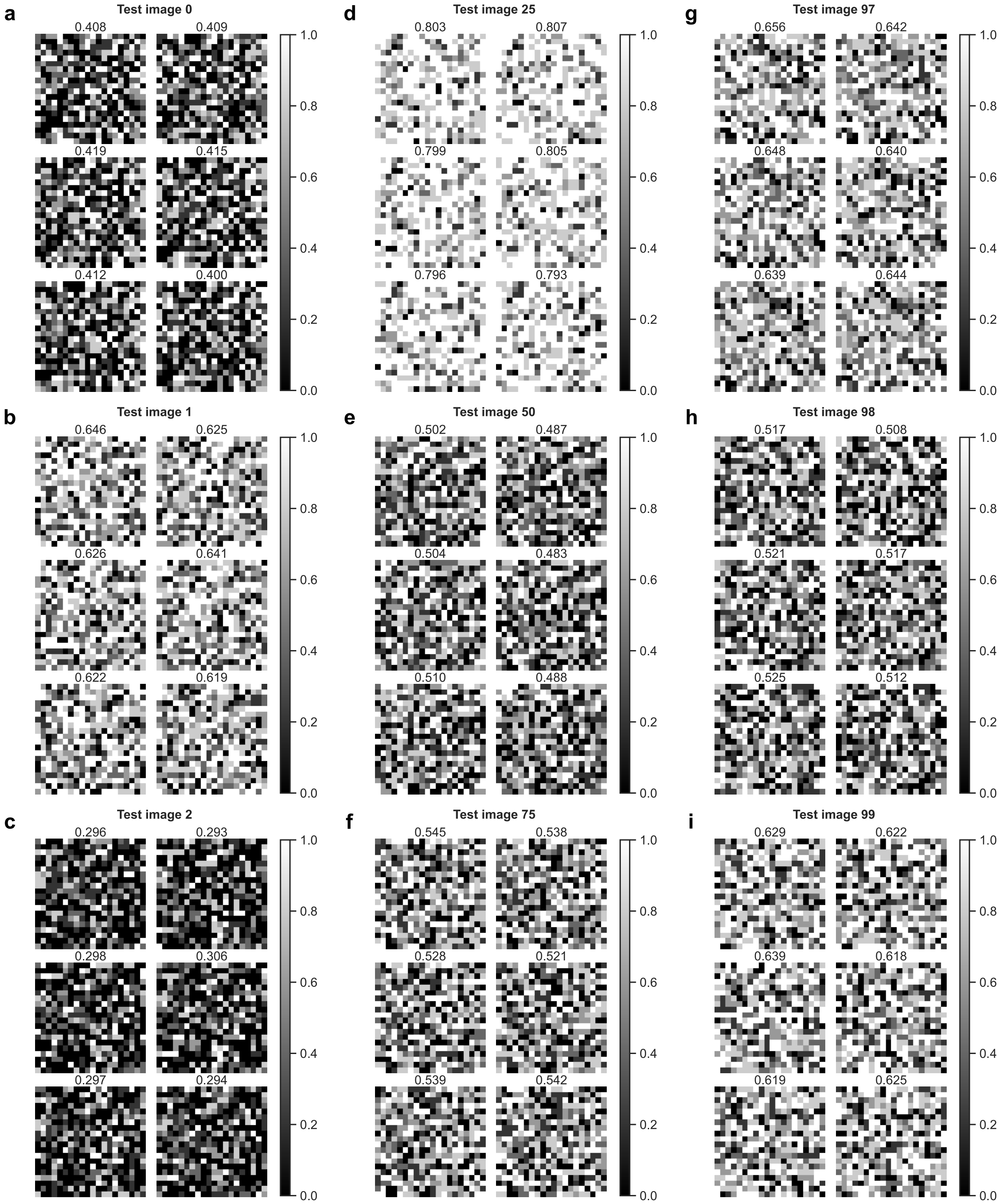

Supplementary Figure 18. Visualization of activation values on the SLM during the last layer experiment. This figure displays the activations obtained from the data collected for the model of $N = 400$ hidden neurons and $K = 5$ shots per activation. The possible values for the 5-shot activations are 0, 0.2, 0.4, 0.6, 0.8, and 1. The activations of size 400 are rearranged into a 20×20 shape, which corresponds to their physical layout on the SLM. Panels **a** to **i** display the activations of test images with indices 0, 1, 2, 25, 50, 75, 97, 98, and 99, respectively, each with 6 repetitions of inference. The average value of the activations in each block is indicated at the top. The overall average activation value of the 100 test images is ~ 0.5219.

Exposure time	Photons per detection (pos.)	Photons per detection (neg.)	Total photons in output layer	Total detected photons in a full inference	Detected photons per MAC	Test accuracy
1.03 ms	79.9 ± 0.10	79.4 ± 0.10	1592.5 ± 1.5	2636.3 (0.98 fJ)	0.008 (0.003 aJ)	$84.7 \pm 3.2\%$
2.06 ms	159.9 ± 0.17	158.2 ± 0.20	3184.7 ± 2.5	4228.4 (1.57 fJ)	0.013 (0.005 aJ)	$92.0 \pm 2.3\%$
3.09 ms	239.7 ± 0.17	237.8 ± 0.22	4777.4 ± 2.6	5821.1 (2.17 fJ)	0.018 (0.007 aJ)	$95.2 \pm 2.0\%$
4.12 ms	320.2 ± 0.21	317.0 ± 0.20	6369.4 ± 2.5	7413.2 (2.76 fJ)	0.023 (0.009 aJ)	$96.4 \pm 1.8\%$
7.21 ms	560.1 ± 0.30	555.2 ± 0.33	11145.7 ± 5.1	12189.5 (4.54 fJ)	0.038 (0.014 aJ)	$98.0 \pm 1.3\%$
12.36 ms	960.1 ± 0.32	950.2 ± 0.40	19107.3 ± 4.1	20151.1 (7.51 fJ)	0.063 (0.024 aJ)	$98.2 \pm 1.1\%$
18.54 ms	1439.3 ± 0.56	1425.8 ± 0.41	28658.1 ± 6.6	29701.8 (11.08 fJ)	0.094 (0.035 aJ)	$99.0 \pm 1.0\%$

Supplementary Table 6. Optical energy consumption in SPDNN inference with varying photon budgets in the optical implementation of the output layer. The first column displays the exposure time of the camera, which determines the number of detected photons. The average photons per detection for both positive (pos.) and negative (neg.) output are calculated from the 6000 dot products derived from 100 input images, 6 repetitions in the first layer inference, and 10 output nodes. The total photons in the output layer are determined by averaging 600 inferences of the last layer, each computing 10 output values. The total detected photons in a full inference are the sum of photons detected in both layers. The average photons per MAC is calculated by dividing the total number of MACs by the total detected photons. Standard deviations are calculated based on 30 repetitions of the last layer detection. The total detected number of photons in a full inference, along with the corresponding optical energy of photons at 532 nm, are displayed in the fifth column, with standard deviations omitted for simplicity. These results add the 1043.7 photons used in the first layer. The sixth column displays the average detected number of photons per MAC during a full inference, dividing the numbers in the fifth column by the total number of MACs, 317,600. The last column shows the test accuracy of the inferences at each photon budget.

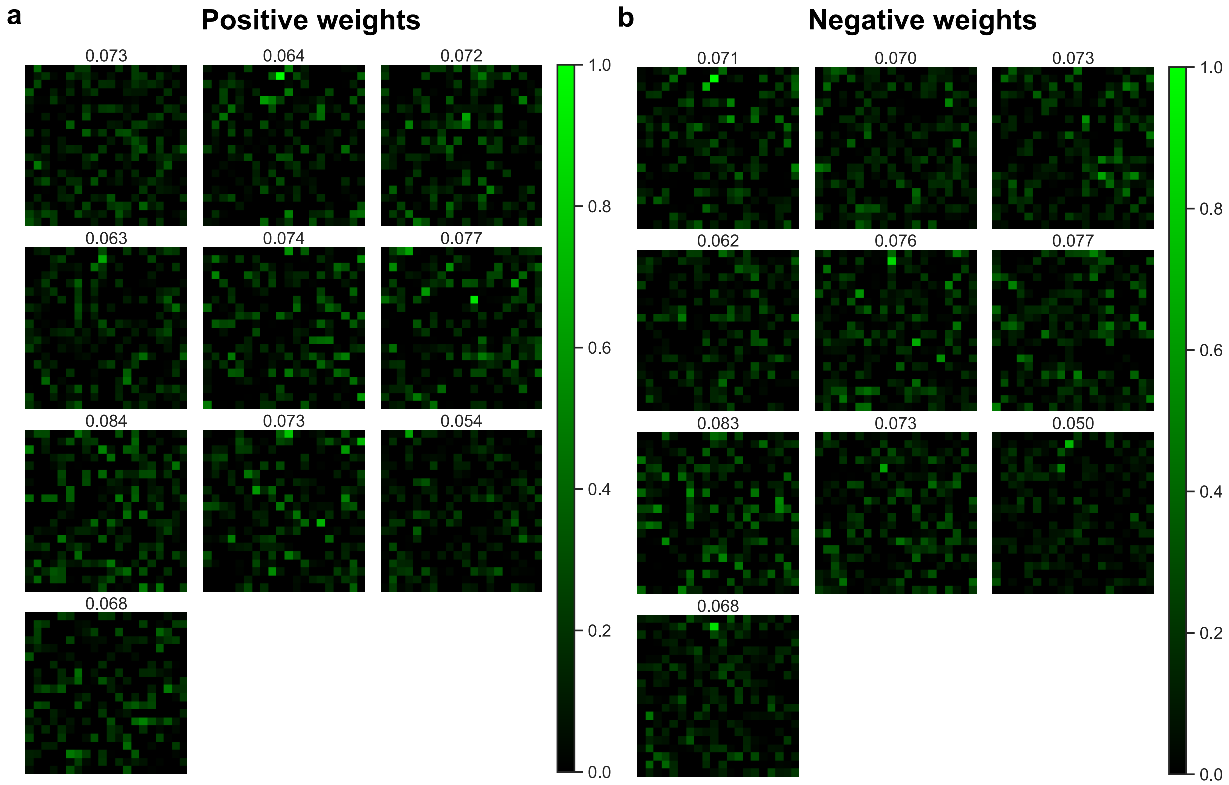

Supplementary Figure 19. Visualization of output layer weights on the OLED display. This figure presents the positive and negative weights from a model with $N = 400$ hidden neurons and a weight matrix of shape 400×10. The positive and negative weights are depicted in panels **a** and **b**, respectively. To match the layout on the OLED display, each weight vector of size 400, corresponding to one of the 10 output nodes, was rearranged into a block with a shape of 20×20 and displayed using green pixels. The values were normalized to the range of 0 to 1 and the average value of each block is indicated at the top.

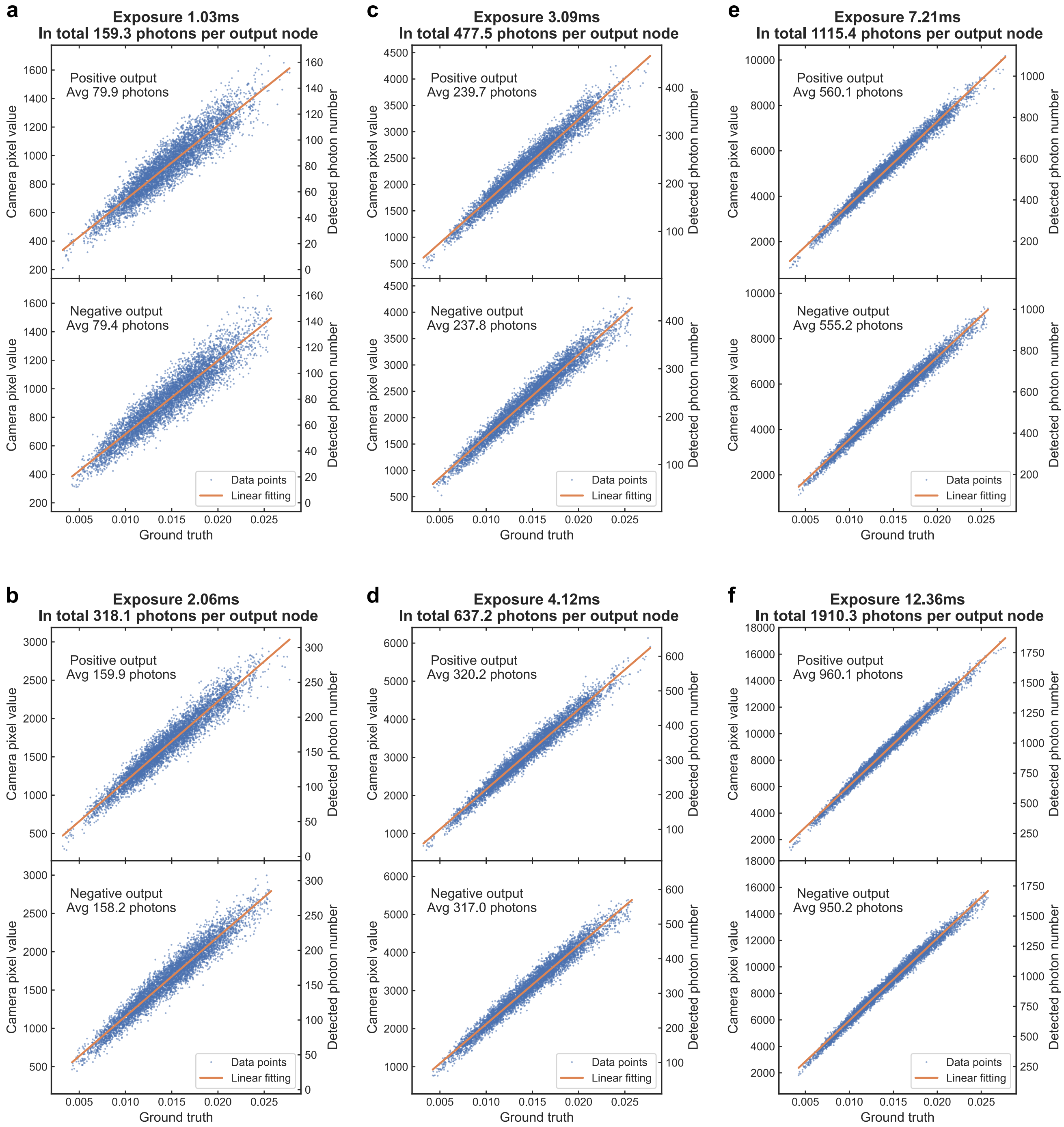

Supplementary Figure 20. Calibration of collected experimental data of the last layer inference. This figure shows the raw data of "high-SNR" optical measurement on the qCMOS camera with various exposure times, each depicted in a separate panel. For each exposure time, one output value was obtained by measuring the output from both positive and negative weights. Each plot includes 6,000 data points, representing 100 test images, 6 repetitions in the hidden layer activation, and 10 output nodes. The ground truth values were computed using full-precision operations on a digital computer. Both the raw camera pixel values and the corresponding detected photon numbers are displayed on the y-axis, with the average detected photon numbers for the 6,000 data points noted in each plot.

we only have one shot per inference. In a full inference with $N = 400$ hidden neurons and $K = 5$ shots per activation, the total number of vector-vector products in the first layer is 400 and that in the last layer is 10 for the 10 output nodes. With dot products of size 784 in the first layer and 400 in the last layer, the total number of MACs in one inference process is equal to 317,600 ($400 \times 784 + 10 \times 400$). To calculate the number of detected photons per MAC, we divide the total number of detected photons in a full inference by the total number of MACs. The prediction of a given inference is made by directly evaluating the output values of each of the 10 output nodes. The output values are calculated as the difference between the positive and negative output intensity. The label of the node with the highest output value is then determined to be the predicted label. The test accuracy on the 100 test images is presented with its mean and standard deviation in the final column of Supplementary Table 6. The standard deviation is determined by considering both the 6 repetitions of the first layer's inference and the 100 repetitions of detections in the last layer.

To visualize the impact of photon noise on accuracy in ONN inferences with a limited photon budget, the data collected from the last layer inference is depicted in Supplementary Figure 20. In each panel, 6000 data points are plotted for either positive or negative output, considering the 100 input images, 6 repetitions in the first layer inference, and 10 output nodes. The ground truth dot product values are computed with high-precision operations on a computer. Both the raw camera pixel values and the corresponding photon count are shown on the vertical axes. As the number of detected photons per detection increases, the detected values become less noisy, resulting in a test accuracy that is closer to the ground truth of 99.2% (Supplementary Table 5). Similar to conventional optical neural networks, the decrease in accuracy is primarily due to shot noise.

In addition, we performed the output layer optically for other configurations as well. The results are represented in Supplementary Figure 21). The activation values collected in experiments of other choices of number of hidden neuron N and shots of SPD readouts K are used as the input for the output layer. If the output layer is implemented with full numerical precision, the test accuracies were shown in Supplementary Table 5. These accuracies are the upper bound for the full-optical implementation with the presence of noise in optical implementation. For these configurations of numbers of hidden neurons (N) and shots of SPD measurements per activation (K), one inference through the $784 \to N$ hidden layer involves $N \times K$ SPD measurements to compute the activation vector in the hidden layer of size N. The detected number of photons for the SPD activation computation in the hidden layer of each configuration is denoted in the corresponding panel in Supplementary Figure 21. The total number of detected photons per inference is the summation of this number and the total number of photons detected in the $N \to 10$ output layer, similar to the procedure we discussed above for the configuration of $N = 400$ and $K = 5$.

Similar to the plot in Figure 3d in the main text, the test accuracies increase with the detected optical energy in a similar trend to that of the $N = 400$, $K = 5$ we discussed in detail above. Comparing these panels with different configurations, we can see that models with a smaller number of neurons N exhibit greater resilience to noise when a similar number of photons are used in the output layer. For instance, comparing panels d and f, with approximately 2000 photons in the output layer (the second point from the left), the test accuracy declines more in the $N = 400$ model (panel f) than in the $N = 100$ model (panel d). Although the models with smaller N and K suffer from a lower noise-free accuracy due to a smaller network size and higher stochasticity, as shown in Supplementary Table 5. The final test accuracy is a combination of these two factors.

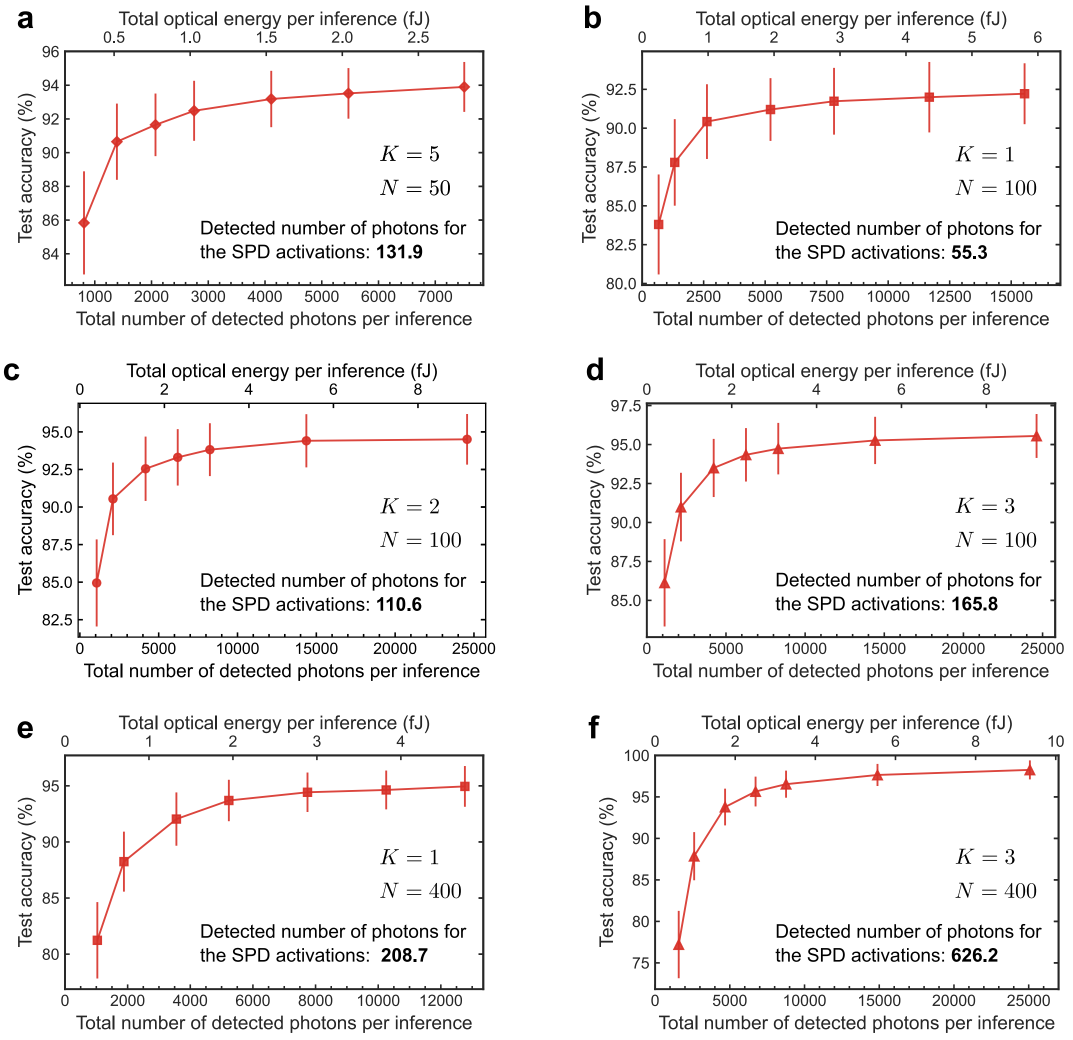

Supplementary Figure 21. Experimental results of full-optical implementation with different SPDNN configurations. In addition to Figure 3d in the main text, this figure shows the results obtained with different numbers of hidden neurons (N) and shots of SPD measurements per activation (K). Each model uses experimentally collected activation values as input for the optical implementation of the output layer. The number of detected photons in the first $784 \to N$ layer to compute the SPD activations in each configuration is denoted in the corresponding plot. The noise-free test accuracies with full-precision output layer are shown in Supplementary Table 5. The number of detected photons in the $N \to 10$ output layer is varied to control the noise in the optical implementation, which is reflected in the resultant test accuracy. The total number of detected photons per inference is the sum of the photon budgets in the two layers.

Part IV

Discussion

Supplementary Note 10. ROBUSTNESS TESTS OF SPDNNS

The first thing to check is the errors induced by the single-photon detectors. The two key parameters to consider when choosing commercial SPDs are photon detection efficiency and dark count rate. Photon detection efficiency refers to the amount of incident light that can be detected by the SPD. Although low photon detection efficiency is a common issue in many photon experiments, it does not add extra noise to our SPDNN models. This is because any attenuation to the light still follows a Poisson distribution and cannot be noisier than a single-photon detector. Hence, a low photon detection efficiency will only add to the overall transmission loss in the setup, and the input light power is usually redundant, so it will not affect the performance much. On the other hand, dark count rates, or false clicks, could pose a greater challenge in experiments with SPDs. False clicks are hard to distinguish from real signals, and the output of the detection is binary. The dark count rate of a functional SPD is typically between 10^{-5} and 10^{-2} false clicks per signal, depending on the experimental configuration. In some extreme circumstances, such as when the exposure time is very long or when it is hard to remove ambient light, the dark count rate could be as high as one false click in tens of detections, ruining the results of the experiment. However, our SPDNN models are resilient to high dark count rates. As shown in Supplementary Figure 22a, even with a false click in fewer than 10 measurements, we still obtain relatively good accuracy. The common range of $< 10^{-2}$ barely affects the performance of the SPDNNs.

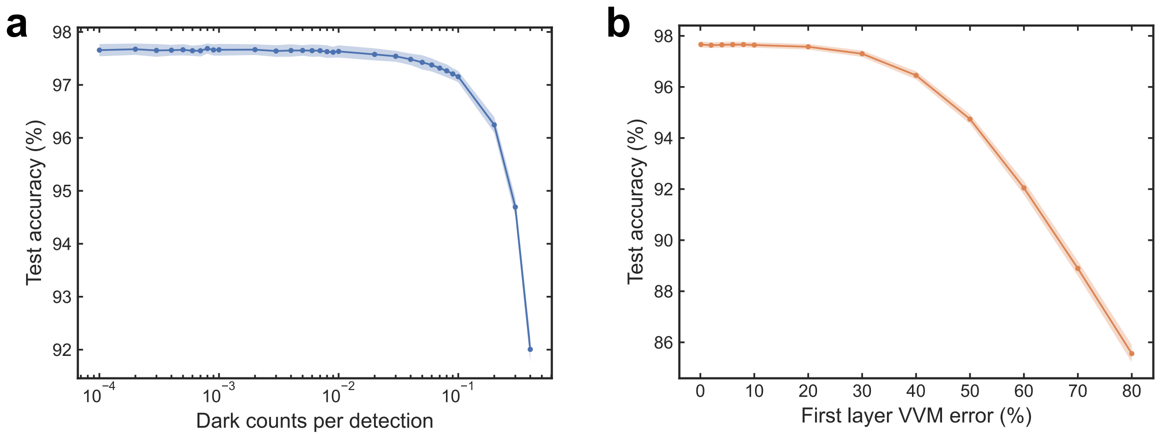

Supplementary Figure 22. Robustness tests for the SPDNN model. a, MNIST test accuracy as a function of dark count rate of each SPD activation measurement. The dark count rate is varied to test the robustness of the SPDNN to noise in the measurement process. **b,** MNIST test accuracy as a function of the relative errors in the vector-vector multiplications (VVMs, or dot products) in the first layer. The errors are introduced at a fixed ratio with respect to the dot product result to simulate systematic errors in the optical setup. Both panels present results obtained from the $784 \to 400 \to 10$ incoherent model with one shot per inference (N = 400, K = 1), and the test accuracies are computed on the full test set of 10,000 images.

As introduced in Supplementary Note 4, the dark count rate with our SPD setting is 0.01 per second per pixel. Given the exposure time of milliseconds, the effects due to dark counts are trivial in the experimental implementation. In summary, the robustness of SPDNN models to noise obviates the need for selecting specialized SPDs for experimental realization. Cost-effective SPDs can be employed for implementing SPDNNs with high performance. Furthermore, considering the significant power consumption of cooling systems for state-of-the-art SPDs, relaxing the dark current requirement can greatly reduce the power consumption of the detection system.

The precision of linear operations is a crucial factor in neural network inferences. As discussed in Supplementary Note 5, the accuracy of vector-vector multiplication may not be optimal when using a single-pixel camera for single-photon detection. To assess the effect of errors in dot product calculations on the performance, we conducted a simulation test by adding different levels of random noise to the dot product results in the first layer, which serve as the pre-activations to the SPD activation function. The results, shown in Supplementary Figure 22b, indicate that SPDNNs are robust to errors in linear operations, even with up to 20% relative noise. This robustness ensures the reliability of the experimental implementation.

Supplementary Note 11. NOISE RESILIENCE COMPARED TO CONVENTIONAL MODELS

In our SPD activation function, two key features set it apart from conventional neural networks: the quantization of activation values and the stochastic activation process. Both of these processes occur naturally through the detection of single photons. The intrinsic quantization of energy and detection process results in a nonlinear response to the input light intensity, eliminating the need for additional nonlinear operations in the neural network. This nonlinearity is evident in the higher MNIST classification test accuracy of SPDNNs compared to linear models. Additionally, the intrinsic photon noise in the activation function makes the output values stochastic. With more averaging, the stochasticity is reduced, resulting in a more precise output as seen in the implementation of SPD activations in the fully-connected layers. This may imply that the noise is unwanted in the neural network inferences. However, the stochastic inference is inevitable in many real-world tasks with a physical device, our stochastic models demonstrated a high noise-resilience that can still yield reliable outputs regardless of this amount of stochasticity.

To evaluate the noise resilience of our SPDNNs against conventional continuous-variable models, we conducted experiments to compare the test accuracy of the models under varying levels of photon noise. We adopted quantization-aware training (QAT) as a popular noise-aware training method, which involves quantizing the weights during training to make the model more noise-resilient. We trained deterministic QAT models with the same multi-layer perceptron (MLP) structure of $784 \to 400 \to 10$ and quantized the weight precision to a specific number of bits. We then compared the MNIST test accuracy of these models to SPDNNs with the same level of photon noise added during the neural network inference of the hidden layer.

For the real-valued QAT models that are compared to the coherent SPDNNs, we chose to use the ReLU activation functions. The QAT models adopted a deterministic quantization function and quantized the weights to the corresponding precision. During inferences, we performed computations with full precision, with the photon noise added to the pre-activation values of the hidden neurons. Supplementary Figure 23a shows that the ReLU models exhibit

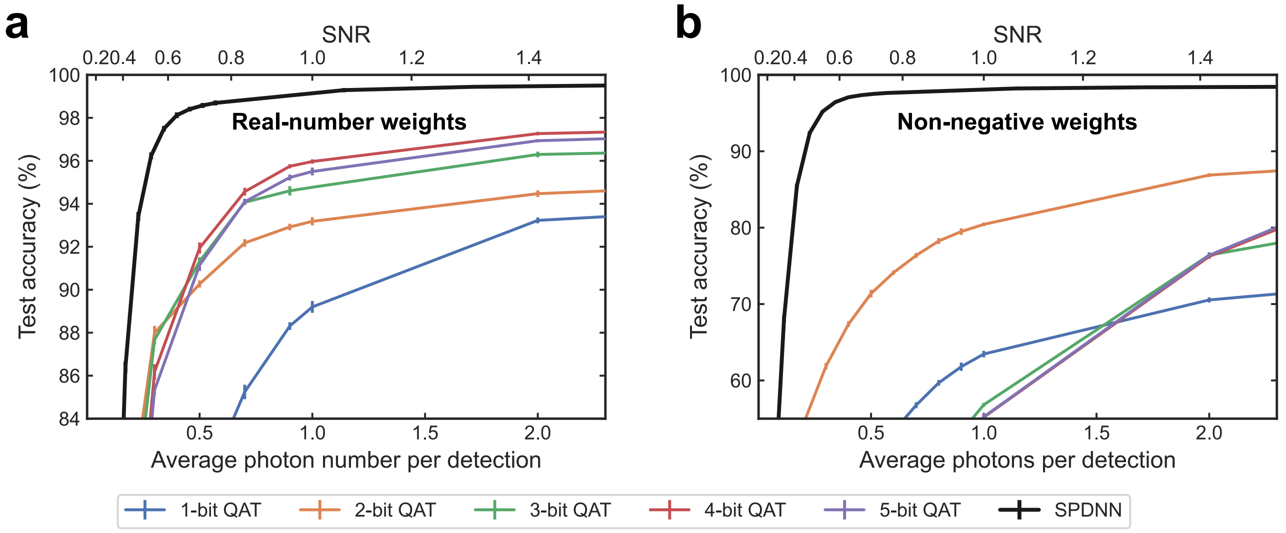

Supplementary Figure 23. Comparison of SPDNNs and QAT models on MNIST classification. a, The test accuracy of MNIST classification using models with real-number weights is shown as a function of photon budget in the hidden layer. The SPDNN model employs the coherent SPD activation function, while the QAT models use ReLU as the activation function. **b,** The test accuracy of MNIST classification using models with non-negative weights is shown as a function of photon budget in the hidden layer. The SPDNN model employs an incoherent setup with non-negative weights, while the QAT models use Sigmoid activation function. The test accuracies are calculated on the full test set of 10,000 images. Both panels present results obtained from the $784 \to 400 \to 10$ model with one shot per inference ($N = 400$, $K = 1$), and the test accuracies are computed on the full test set of 10,000 images.

high noise resilience, and harsh quantization does not significantly enhance the noise resilience but harms the overall precision. In fact, decreasing the quantization levels leads to decreased model performance at this photon noise level. The accuracy almost converges at a precision of 5 bits or higher.

For the non-negative QAT models that are compared to the incoherent SPDNNs, the non-negativity of the weights renders ReLU activation functions less effective. Hence, we use the Sigmoid activation function, more rigorously, the positive half of it, to train the QAT models. However, the models are not as noise resilient as with real-number operations, and stronger quantization is required to enhance the model robustness. As the simulation results show, the performance of models of precision 3 bits or more almost converges. It is worth noting that, despite having over 98% test accuracy without photon noise, the performance of these models with 3-bit precision or more is worse under such noise levels. Decreasing the quantized precision is a tradeoff between noise resilience and overall accuracy. We observed that the 2-bit QAT model performs the best over other precisions. These results showed that all the QAT models are inferior to SPDNNs in terms of accuracy under the same or lower photon budget. This finding indicates that SPDNNs are more effective in achieving high accuracy in photon-starved environments.

Our results suggest that natural quantization of optical energy enhances noise resilience in neural networks, and that stochasticity could aid in searching for more accurate and noise-resilient models. However, we do not claim that the SPD activation function is the best way to train a noisy neural network, and we are open to exploring other noise-aware training methods that could further improve resilience. Our findings demonstrate that with appropriate training that takes into account the stochastic and quantized nature of optical energy in the realistic physical computing system, ONNs can achieve high performance even at very high noise levels, which was not previously possible. What makes it more intriguing about our approach is that it exploits the natural single-photon detection process.

Supplementary Note 12. DISTRIBUTION OF EXPECTATION VALUES FOR SPD ACTIVATIONS

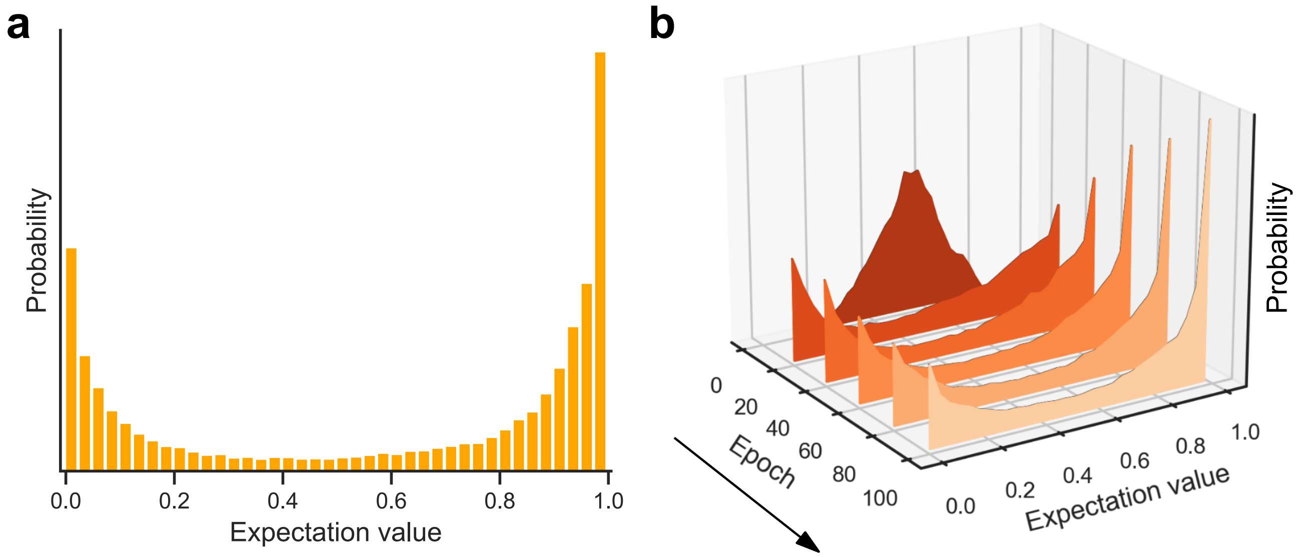

Supplementary Figure 24. Distribution of expectation values for hidden neuron activations. a, The distribution of expectation values for each hidden neuron during the inference of a trained model. The model follows a multi-layer perceptron (MLP) structure of $784 \to 400 \to 400 \to 10$ using a coherent SPD activation function and real-valued weights (Supplementary Note 2). The expectation values of neuron activations in the first hidden layer are depicted. **b,** The evolution of the expectation value distribution during training. Different expectation value distributions of hidden neuron activations (as in **a**) are plotted at six training epochs to demonstrate changes throughout the training process.

In this study, we explored the use of highly stochastic SPDNN models to achieve high performance in deterministic classification tasks. At first glance, this may seem counter-intuitive, as deterministic classification typically requires stable and reliable outputs, while stochastic models introduce inherent uncertainty. However, a closer examination of the characteristics of the activation values in SPDNN inferences provides a more intuitive understanding of how this approach can achieve such high accuracy.

In Supplementary Figure 24a, we present the distribution of expectation values for hidden neuron activations. This distribution is obtained using a single shot of SPD readout ($K = 1$). Since the activations are binary (either 0 or 1), the expectation value represents the probability of the activation being 1. We constructed this histogram by considering the inferences for all input images in the test set and all hidden neurons' activation values, so that the distribution is averaged over many different samples to show the overall picture of the general behavior of the network inference. For example, a layer with 400 hidden neurons and 10,000 test input images would yield $400 \times 10,000 = 4 \times 10^6$ expectation values included in the histogram. We utilized an optimized SPDNN model with an MLP structure of $784 \to 400 \to 400 \to 10$ to generate this histogram, and we also found that this distribution is consistent across models with varying numbers of hidden neurons or layers, as well as coherent or incoherent SPD detection schemes.

Interestingly, we observed that the majority of neuron activations exhibit more deterministic expectation values rather than pure randomness. While some models trained with experimental limitations cannot reach absolute zero values, the peak at zero value shifts to a less sharp bump close to zero, still distributing towards either end rather than the middle value of 0.5. In Bernoulli sampling, an expectation value of 0.5 signifies that the probability of being 0 or 1

is equivalent, indicating that there is no useful information in the process, and the entropy is at its maximum. Noisy channels with such characteristics cannot carry valuable information for neural network inference. Consequently, during the training process, the model should strive to learn from the training set and update the neural network weights accordingly to capture the essential features. This process involves storing information in the trained model, which can be reflected by decreasing the entropy of each stochastic binary neuron.

In Supplementary Figure 24b, we observe that as the model undergoes more training epochs, the expectation value distribution of activations becomes more concentrated towards 0 or 1. This indicates that the model retains more information and generates more reliable outputs.

However, it is important to note that while the entropy of each individual neuron decreases, at the network level, the average activation still tends to be around 0.5 photons when considering all the neurons, denoting maximum entropy. This suggests that the neural network is effectively utilizing its capacity to extract information using all its neurons by increasing the overall network entropy. In fact, a network with all neurons having the same expectation value (entropy of 0) would not be able to learn any meaningful features.

In summary, while SPDNNs are inherently stochastic, the distribution of expectation values for hidden neuron activations leans towards deterministic outcomes, allowing the model to effectively learn features and achieve high accuracy in deterministic classification tasks. The training process shapes the probabilistic distribution of the neurons and allocates different neurons close to either 0 or 1 to learn the patterns of input images and output reliable inferences. Remarkably, the implementation of this allocation is exceptionally efficient in optical energy, as each activation only involves a photon click.

Supplementary Note 13. BACKGROUND FOR STOCHASTIC COMPUTING

The stochastic operation of neural networks has been extensively studied in computer science as part of the broader field of stochastic computing [35]. In the field of machine learning, binary stochastic neurons (BSNs) have been used to construct stochastic neural networks [1, 5, 9, 31, 36–38], with training being a major focus of study. Investigations of hardware implementations of stochastic computing neural networks, such as those in Refs. [2, 39] (with many more surveyed in Ref. [40]), have typically been for deterministic complementary metal–oxide–semiconductor (CMOS) electronics, with the stochasticity introduced by random-number generators. While many studies of binary stochastic neural networks have been conducted with standard digital CMOS processors, there have also been proposals to construct them from beyond-CMOS hardware, motivated by the desire to minimize power consumption: direct implementation of binary stochastic neurons using bistable systems that are noisy by design—such as low-barrier magnetic tunnel junctions (MTJs)—has been explored [41–43], and there have also been proposals to realize hardware stochastic elements for neural networks that could be constructed with noisy CMOS electronics or other physical substrates [44, 45]. ONNs in which noise has been intentionally added [46–48] have also been studied. Our work with low-photon-count optics is related but distinct from many of the studies cited here in its motivating assumption: instead of desiring noise and stochastic behavior—and purposefully designing devices to have them, we are concerned with situations in which physical devices have large and unavoidable noise but where we would like to nevertheless construct deterministic classifiers using these devices because of their potential for low-energy computing (Fig. 1 in main text).

Supplementary Note 14. PROSPECTS FOR FUTURE APPLICATIONS

While we have demonstrated a fundamental point—that ONNs can be successfully operated in the few-photon-per-activation regime in which quantum shot noise causes very low SNR—an important practical consideration for the construction of ONNs is that the energy used by optical signals within the ONN is only part of the ONN's total energy consumption, and it is the total energy per inference that is generally what one wants to optimize for [49–51]. A practical limitation of our experiments is that they were conducted with a relatively slow[1] single-photon-detector array, limiting the speed at which a single execution of a layer could be carried out, and the detector array was not optimized for energy efficiency. For our fundamental approach and methods to be applied to make ONNs that offer a practical advantage over state-of-the-art electronic processors as generic neural-network accelerators, there remains important work to be done in engineering an overall system that operates sufficiently fast while minimizing total energy cost. Recent progress in the development of large, fast arrays of single-photon detectors coupled with digital logic [52] suggest that there is a path towards this goal. Ref. [53] has also pointed out the possibility of using fast superconducting-nanowire single-photon detectors for realizing spiking neural networks. Furthermore, there is a complementary path toward utility in the nearer term: if instead of aiming to use ONNs to entirely replace electronic processors, one uses ONNs as a pre-processor for input data that is already optical [54–56], operating the ONN with single-photon detectors is a natural match with scenarios in which the optical input is very weak—for example, in low-light-imaging applications.

Our approach is not tied to a specific architecture of ONN—the free-space matrix-vector multiplier used in our experiments is just one of many possible choices of architecture. Other ONNs could be adapted to use our approach by replacing the photodetectors typically used for readout of neurons at the end of a layer with single-photon detectors. ONNs based on diffractive optics [20, 57, 58], Mach-Zehnder interferometer (MZI) meshes [17, 19, 59], and other on-chip approaches to matrix-vector multiplication [22, 23, 60] all appear compatible.

In our optical experiments, we used single-photon detectors that output an electronic signal when a photon is detected. However, in multilayer ONNs, the input to each layer is optical. One can convert an electronic detector output to an optical input by modulating an optical source—which is what we did and what is often done in ONNs more generally [54]—but an alternative is to construct a device that performs SPD with high efficiency and gives the measurement result as an *optical* signal that can be directly used as an input to the next layer in the ONN. Designing and demonstrating such a device is an interesting potential avenue for future work in applied quantum nonlinear optics [61–66], and could lead to both lower electronic energy consumption and higher speed for single-photon-detection ONNs.

We trained our demonstration SPDNN *in silico* using backpropagation, but if SPDNNs with high overall energy efficiency are built, it would be a boon use this efficient hardware not only for inference but also for training. To this end, it could be interesting to study how to adapt *in situ* training [67–70], including backpropagation-free (e.g., Refs. [71–74]), methods for SPDNNs. An open question related to training is whether it is possible to make SPDNNs that do not involve a final high-SNR layer while preserving task accuracy; this could help to reduce the overall energy per inference. Other future work could explore the extension of our research to neural networks with larger sizes (wider

[1] 19.8 kHz maximum frame rate.

and more layers, which could both improve the capability of the neural network and further amortize the energy cost of the final, high-SNR layer, if used), more sophisticated classification tasks (beyond MNIST and CIFAR-10 image classification—such as has been shown with conventional binary neural networks [32, 33, 75]), and generative or other probabilistic tasks—for which the stochasticity can be harnessed rather than merely tolerated. Beyond machine-learning tasks, an SPDNN layer could be used as the core of a single-photon-regime photonic Ising machine [76] for heuristically solving combinatorial-optimization problems, realizing an optical version of p-bit computing [43].

The phenomena observed in our work seemingly relies on two key physical ingredients. First, the system's available states are effectively quantized, as in the photonic quantization of energy in our ONN demonstration, or the binarization that occurs in low-barrier, stochastic magnetic tunnel junctions [77]. Second, the noise in the system results in the quantized outputs of the system being stochastic. This suggests that ultra-low-SNR physical neural networks should be possible in many physical hardware platforms beyond photonics. Systems in which shot noise dominates are natural matches with our approach and methods. Our approach could also be relevant to systems in which thermal (Johnson) noise dominates—as is typically the case in room-temperature electronics—but this will depend on not just the noise but also the system's dynamics. Which hardware platforms and system architectures can yield an overall energy benefit by being operated in a stochastic regime while maintaining computational accuracy is an important open question.

[1] R. M. Neal, Learning stochastic feedforward networks. Department of Computer Science, University of Toronto **64**, 1577 (1990).

[2] V. T. Lee, A. Alaghi, J. P. Hayes, V. Sathe, and L. Ceze, Energy-efficient hybrid stochastic-binary neural networks for near-sensor computing. In Design, Automation & Test in Europe Conference & Exhibition (DATE), 2017, 13–18 (2017).

[3] Y. Liu, S. Liu, Y. Wang, F. Lombardi, and J. Han, A stochastic computational multi-layer perceptron with backward propagation. IEEE Transactions on Computers **67**, 1273–1286 (2018).

[4] G. E. Hinton, T. J. Sejnowski, and D. H. Ackley, (1984) Boltzmann machines: Constraint satisfaction networks that learn. (Carnegie-Mellon University, Department of Computer Science Pittsburgh, PA).

[5] D. H. Ackley, G. E. Hinton, and T. J. Sejnowski, A learning algorithm for Boltzmann machines. Cognitive Science **9**, 147–169 (1985).

[6] R. J. Williams, Simple statistical gradient-following algorithms for connectionist reinforcement learning. Machine learning **8**, 229–256 (1992).

[7] L. Weaver and N. Tao, The optimal reward baseline for gradient-based reinforcement learning. arXiv:1301.2315 (2013).

[8] I. R. Fiete and H. S. Seung, Gradient learning in spiking neural networks by dynamic perturbation of conductances. Physical Review Letters **97**, 048104 (2006).

[9] Y. Bengio, N. Léonard, and A. Courville, Estimating or propagating gradients through stochastic neurons for conditional computation. arXiv:1308.3432 (2013).

[10] G. Hinton, Neural netowrks for machine learning. Coursera, Video Lectures (2012).

[11] P. Yin, J. Lyu, S. Zhang, S. Osher, Y. Qi, and J. Xin, Understanding straight-through estimator in training activation quantized neural nets. arXiv:1903.05662 (2019).

[12] J. Chung, S. Ahn, and Y. Bengio, Hierarchical multiscale recurrent neural networks. arXiv:1609.01704 (2016).

[13] L. Bottou, Stochastic gradient descent tricks. Neural Networks: Tricks of the Trade: Second Edition pp. 421–436 (2012).

[14] D. P. Kingma and J. Ba, Adam: A method for stochastic optimization. arXiv:1412.6980 (2014).

[15] I. Loshchilov and F. Hutter, Decoupled weight decay regularization. arXiv:1711.05101 (2017).

[16] A. Sludds, S. Bandyopadhyay, Z. Chen, Z. Zhong, J. Cochrane, L. Bernstein, D. Bunandar, P. B. Dixon, S. A. Hamilton, M. Streshinsky et al. Delocalized photonic deep learning on the internet's edge. Science **378**, 270–276 (2022).

[17] Y. Shen, N. C. Harris, S. Skirlo, M. Prabhu, T. Baehr-Jones, M. Hochberg, X. Sun, S. Zhao, H. Larochelle, D. Englund et al. Deep learning with coherent nanophotonic circuits. Nature Photonics **11**, 441 (2017).

[18] M. Miscuglio, Z. Hu, S. Li, J. K. George, R. Capanna, H. Dalir, P. M. Bardet, P. Gupta, and V. J. Sorger, Massively parallel amplitude-only Fourier neural network. Optica **7**, 1812–1819 (2020).

[19] W. Bogaerts, D. Pérez, J. Capmany, D. A. B. Miller, J. Poon, D. Englund, F. Morichetti, and A. Melloni, Programmable photonic circuits. Nature **586**, 207–216 (2020).

[20] X. Lin, Y. Rivenson, N. T. Yardimci, M. Veli, Y. Luo, M. Jarrahi, and A. Ozcan, All-optical machine learning using diffractive deep neural networks. Science **361**, 1004–1008 (2018).

[21] J. Spall, X. Guo, T. D. Barrett, and A. Lvovsky, Fully reconfigurable coherent optical vector–matrix multiplication. Optics Letters **45**, 5752–5755 (2020).

[22] J. Feldmann, N. Youngblood, M. Karpov, H. Gehring, X. Li, M. Stappers, M. Le Gallo, X. Fu, A. Lukashchuk, A. S. Raja et al. Parallel convolutional processing using an integrated photonic tensor core. Nature **589**, 52–58 (2021).

[23] X. Xu, M. Tan, B. Corcoran, J. Wu, A. Boes, T. G. Nguyen, S. T. Chu, B. E. Little, D. G. Hicks, R. Morandotti, A. Mitchell, and D. J. Moss, 11 TOPS photonic convolutional accelerator for optical neural networks. Nature **589**, 44–51

(2021).

[24] P. Ramachandran, B. Zoph, and Q. V. Le, Searching for activation functions. arXiv:1710.05941 (2017).

[25] A. Krizhevsky, Learning multiple layers of features from tiny images. Technical Report pp. 32–33 (2009).

[26] T. Clanuwat and et al., Deep Learning for Classical Japanese Literature. arXiv preprint arXiv:1812.01718 (2018).

[27] H. Xiao and et al., Fashion-MNIST: a Novel Image Dataset for Benchmarking Machine Learning Algorithms. arXiv preprint arXiv:1708.07747 (2017).

[28] T. Wang, S.-Y. Ma, L. G. Wright, T. Onodera, B. C. Richard, and P. L. McMahon, An optical neural network using less than 1 photon per multiplication. Nature Communications **13**, 1–8 (2022).

[29] K. Dhimitri, S. M. Fullerton, B. Coyle, K. E. Bennett, T. Miura, T. Higuchi, and T. Maruno, Scientific CMOS (sCMOS) camera capabilities with a focus on quantum applications. In Photonics for Quantum 2022, PC122430L (2022).

[30] C. Wu, X. Yang, H. Yu, R. Peng, I. Takeuchi, Y. Chen, and M. Li, Harnessing Optoelectronic Noises in a Hybrid Photonic Generative Adversarial Network (GAN). Preprint at Research Square:10.21203 (2021).

[31] I. Hubara, M. Courbariaux, D. Soudry, R. El-Yaniv, and Y. Bengio, Binarized neural networks. Advances in Neural Information Processing Systems **29** (2016).

[32] M. Rastegari, V. Ordonez, J. Redmon, and A. Farhadi, XNOR-Net: Imagenet classification using binary convolutional neural networks. In Computer Vision–ECCV 2016: 14th European Conference, Amsterdam, The Netherlands, October 11–14, 2016, Proceedings, Part IV, 525–542 (2016).

[33] A. Bulat, J. Kossaifi, G. Tzimiropoulos, and M. Pantic, Matrix and tensor decompositions for training binary neural networks. arXiv:1904.07852 (2019).

[34] H. Qin, R. Gong, X. Liu, X. Bai, J. Song, and N. Sebe, Binary neural networks: A survey. Pattern Recognition **105**, 107281 (2020).

[35] A. Alaghi and J. P. Hayes, Survey of stochastic computing. ACM Transactions on Embedded Computing Systems (TECS) **12**, 1–19 (2013).

[36] R. M. Neal, Connectionist learning of belief networks. Artificial Intelligence **56**, 71–113 (1992).

[37] C. Tang and R. R. Salakhutdinov, Learning stochastic feedforward neural networks. Advances in Neural Information Processing Systems **26** (2013).

[38] T. Raiko, M. Berglund, G. Alain, and L. Dinh, Techniques for learning binary stochastic feedforward neural networks. arXiv:1406.2989 (2014).

[39] Y. Ji, F. Ran, C. Ma, and D. J. Lilja, A hardware implementation of a radial basis function neural network using stochastic logic. In 2015 Design, Automation & Test in Europe Conference & Exhibition (DATE), 880–883 (2015).

[40] Y. Liu, S. Liu, Y. Wang, F. Lombardi, and J. Han, A survey of stochastic computing neural networks for machine learning applications. IEEE Transactions on Neural Networks and Learning Systems **32**, 2809–2824 (2020).

[41] D. Vodenicarevic, N. Locatelli, A. Mizrahi, J. S. Friedman, A. F. Vincent, M. Romera, A. Fukushima, K. Yakushiji, H. Kubota, S. Yuasa et al. Low-energy truly random number generation with superparamagnetic tunnel junctions for unconventional computing. Physical Review Applied **8**, 054045 (2017).

[42] O. Hassan, R. Faria, K. Y. Camsari, J. Z. Sun, and S. Datta, Low-barrier magnet design for efficient hardware binary stochastic neurons. IEEE Magnetics Letters **10**, 1–5 (2019).

[43] S. Chowdhury, A. Grimaldi, N. A. Aadit, S. Niazi, M. Mohseni, S. Kanai, H. Ohno, S. Fukami, L. Theogarajan, G. Finocchio et al. A full-stack view of probabilistic computing with p-bits: devices, architectures and algorithms. IEEE Journal on Exploratory Solid-State Computational Devices and Circuits (2023).

[44] T. Hylton, T. M. Conte, and M. D. Hill, A vision to compute like nature: Thermodynamically. Communications of the ACM **64**, 35–38 (2021).

[45] P. J. Coles, C. Szczepanski, D. Melanson, K. Donatella, A. J. Martinez, and F. Sbahi, Thermodynamic AI and the

fluctuation frontier. arXiv:2302.06584 (2023).

[46] C. Wu, X. Yang, H. Yu, R. Peng, I. Takeuchi, Y. Chen, and M. Li, Harnessing optoelectronic noises in a photonic generative network. Science Advances **8**, eabm2956 (2022).

[47] C. Wu, X. Yang, Y. Chen, and M. Li, Photonic Bayesian neural network using programmed optical noises. IEEE Journal of Selected Topics in Quantum Electronics **29**, 1–6 (2022).

[48] B. Ma, J. Zhang, X. Li, and W. Zou, Stochastic photonic spiking neuron for Bayesian inference with unsupervised learning. Optics Letters **48**, 1411–1414 (2023).

[49] M. A. Nahmias, T. F. De Lima, A. N. Tait, H.-T. Peng, B. J. Shastri, and P. R. Prucnal, Photonic multiply-accumulate operations for neural networks. IEEE Journal of Selected Topics in Quantum Electronics **26**, 1–18 (2019).

[50] R. Hamerly, L. Bernstein, A. Sludds, M. Soljačić, and D. Englund, Large-scale optical neural networks based on photoelectric multiplication. Physical Review X **9**, 021032 (2019).

[51] M. G. Anderson, S.-Y. Ma, T. Wang, L. G. Wright, and P. L. McMahon, Optical transformers. arXiv:2302.10360 (2023).

[52] C. Bruschini, S. Burri, E. Bernasconi, T. Milanese, A. C. Ulku, H. Homulle, and E. Charbon, LinoSPAD2: A 512x1 linear SPAD camera with system-level 135-ps SPTR and a reconfigurable computational engine for time-resolved single-photon imaging. In Quantum Sensing and Nano Electronics and Photonics XIX Vol. 12430, 126–135 (2023).

[53] J. M. Shainline, S. M. Buckley, R. P. Mirin, and S. W. Nam, Superconducting optoelectronic circuits for neuromorphic computing. Physical Review Applied **7**, 034013 (2017).

[54] G. Wetzstein, A. Ozcan, S. Gigan, S. Fan, D. Englund, M. Soljačić, C. Denz, D. A. Miller, and D. Psaltis, Inference in artificial intelligence with deep optics and photonics. Nature **588**, 39–47 (2020).

[55] T. Wang, M. M. Sohoni, L. G. Wright, M. M. Stein, S.-Y. Ma, T. Onodera, M. G. Anderson, and P. L. McMahon, Image sensing with multilayer nonlinear optical neural networks. Nature Photonics **17**, 408–415 (2023).

[56] L. Huang, Q. A. Tanguy, J. E. Froch, S. Mukherjee, K. F. Bohringer, and A. Majumdar, Photonic Advantage of Optical Encoders. arXiv:2305.01743 (2023).

[57] J. Chang, V. Sitzmann, X. Dun, W. Heidrich, and G. Wetzstein, Hybrid optical-electronic convolutional neural networks with optimized diffractive optics for image classification. Scientific Reports **8**, 12324 (2018).

[58] T. Zhou, X. Lin, J. Wu, Y. Chen, H. Xie, Y. Li, J. Fan, H. Wu, L. Fang, and Q. Dai, Large-scale neuromorphic optoelectronic computing with a reconfigurable diffractive processing unit. Nature Photonics **15**, 367–373 (2021).

[59] J. Carolan, C. Harrold, C. Sparrow, E. Martín-López, N. J. Russell, J. W. Silverstone, P. J. Shadbolt, N. Matsuda, M. Oguma, M. Itoh et al. Universal linear optics. Science **349**, 711–716 (2015).

[60] A. N. Tait, J. Chang, B. J. Shastri, M. A. Nahmias, and P. R. Prucnal, Demonstration of WDM weighted addition for principal component analysis. Optics Express **23**, 12758–12765 (2015).

[61] I. Mazets and G. Kurizki, Multiatom cooperative emission following single-photon absorption: Dicke-state dynamics. Journal of Physics B: Atomic, Molecular and Optical Physics **40**, F105 (2007).

[62] D. Pinotsi and A. Imamoglu, Single photon absorption by a single quantum emitter. Physical Review Letters **100**, 093603 (2008).

[63] F. Sotier, T. Thomay, T. Hanke, J. Korger, S. Mahapatra, A. Frey, K. Brunner, R. Bratschitsch, and A. Leitenstorfer, Femtosecond few-fermion dynamics and deterministic single-photon gain in a quantum dot. Nature Physics **5**, 352–356 (2009).

[64] A. H. Kiilerich and K. Mølmer, Input-output theory with quantum pulses. Physical Review Letters **123**, 123604 (2019).

[65] Q. Li, K. Orcutt, R. L. Cook, J. Sabines-Chesterking, A. L. Tong, G. S. Schlau-Cohen, X. Zhang, G. R. Fleming, and K. B. Whaley, Single-photon absorption and emission from a natural photosynthetic complex. Nature pp. 1–5 (2023).

[66] C. Roques-Carmes, Y. Salamin, J. Sloan, S. Choi, G. Velez, E. Koskas, N. Rivera, S. E. Kooi, J. D. Joannopoulos, and M. Soljačić, Biasing the quantum vacuum to control macroscopic probability distributions. Science **381**, 205–209 (2023).

[67] T. Zhou, L. Fang, T. Yan, J. Wu, Y. Li, J. Fan, H. Wu, X. Lin, and Q. Dai, In situ optical backpropagation training of diffractive optical neural networks. Photonics Research **8**, 940–953 (2020).

[68] X. Guo, T. D. Barrett, Z. M. Wang, and A. Lvovsky, Backpropagation through nonlinear units for the all-optical training of neural networks. Photonics Research **9**, B71–B80 (2021).

[69] S. Bandyopadhyay, A. Sludds, S. Krastanov, R. Hamerly, N. Harris, D. Bunandar, M. Streshinsky, M. Hochberg, and D. Englund, Single chip photonic deep neural network with accelerated training. arXiv:2208.01623 (2022).

[70] S. Pai, Z. Sun, T. W. Hughes, T. Park, B. Bartlett, I. A. Williamson, M. Minkov, M. Milanizadeh, N. Abebe, F. Morichetti et al. Experimentally realized in situ backpropagation for deep learning in photonic neural networks. Science **380**, 398–404 (2023).

[71] Y. Bengio, D.-H. Lee, J. Bornschein, T. Mesnard, and Z. Lin, Towards biologically plausible deep learning. arXiv:1502.04156 (2015).

[72] T. P. Lillicrap, A. Santoro, L. Marris, C. J. Akerman, and G. Hinton, Backpropagation and the brain. Nature Reviews Neuroscience **21**, 335–346 (2020).

[73] G. Hinton, The concept of mortal computation. Keynote address presented at the Neural Information Processing Systems conference, New Orleans (2023).

[74] M. Stern and A. Murugan, Learning without neurons in physical systems. Annual Review of Condensed Matter Physics **14**, 417–441 (2023).

[75] A. Bulat and G. Tzimiropoulos, XNOR-Net++: Improved binary neural networks. arXiv:1909.13863 (2019).

[76] N. Mohseni, P. L. McMahon, and T. Byrnes, Ising machines as hardware solvers of combinatorial optimization problems. Nature Reviews Physics **4**, 363–379 (2022).

[77] J. Grollier, D. Querlioz, K. Camsari, K. Everschor-Sitte, S. Fukami, and M. D. Stiles, Neuromorphic spintronics. Nature Electronics **3**, 360–370 (2020).
